# Modulating proton diffusion and conductivity in metal–organic frameworks by incorporation of accessible free carboxylic acid groups†
†Electronic supplementary information (ESI) available. CCDC 1849703–1849705. For ESI and crystallographic data in CIF or other electronic format see DOI: 10.1039/c8sc03022g


**DOI:** 10.1039/c8sc03022g

**Published:** 2018-11-02

**Authors:** Peter Rought, Christopher Marsh, Simona Pili, Ian P. Silverwood, Victoria García Sakai, Ming Li, Martyn S. Brown, Stephen P. Argent, Inigo Vitorica-Yrezabal, George Whitehead, Mark R. Warren, Sihai Yang, Martin Schröder

**Affiliations:** a School of Chemistry , University of Manchester , Manchester M13 9PL , UK . Email: Sihai.Yang@manchester.ac.uk ; Email: M.Schroder@manchester.ac.uk; b ISIS Pulsed Neutron and Muon Source , Rutherford Appleton Laboratory , Oxfordshire OX11 0QX , UK; c School of Engineering , University of Nottingham , Nottingham NG7 2RD , UK; d School of Chemistry , University of Warwick , Coventry CV4 7AL , UK; e Diamond Light Source , Harwell Science and Innovation Campus , Oxfordshire OX11 0DE , UK

## Abstract

Three multi-carboxylic acid functionalised ligands have been designed, synthesised and utilised to prepare the new barium-based MOFs, MFM-510, -511, and -512, which show excellent stability to water-vapour. MFM-510 and MFM-511 show moderate proton conductivities (2.1 × 10^–5^ and 5.1 × 10^–5^ S cm^–1^, respectively) at 99% RH and 298 K, attributed to the lack of free protons or hindered proton diffusion within the framework structures. In contrast, MFM-512, which incorporates a pendant carboxylic acid group directed into the pore of the framework, shows a two orders of magnitude enhancement in proton conductivity (2.9 × 10^–3^ S cm^–1^). Quasi-elastic neutron scattering (QENS) suggests that the proton dynamics of MFM-512 are mediated by “free diffusion inside a sphere” confirming that incorporation of free carboxylic acid groups within the pores of MOFs is an efficient albeit synthetically challenging strategy to improve proton conductivity.

## Abstract 2

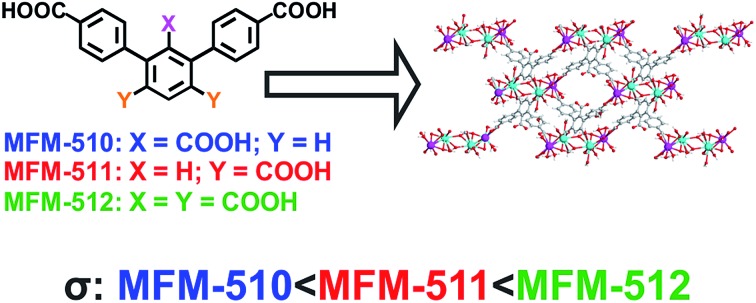
The proton conductivity of barium-based MOFs MFM-510 to MFM-512 are analysed in relation to the absence and presence of free –COOH groups in the pores.

## Introduction

Proton exchange membrane fuel cells (PEMFCs) are a promising technology for the use of hydrogen-based energy in applications such as transport.^1^ A key factor for its success is the development of materials which can move protons efficiently across the cell. The most commonly used PEM material is the polymer Nafion, which consists of a perfluorinated polyethylene backbone with sulfonic acid terminated side chains.^2^ The acidic nature of this polymer facilitates proton conduction in the order of 10^–2^ S cm^–1^ below 85 °C in the presence of water. However, the lack of long range order in polymers restricts the in-depth understanding of the mechanism of proton conductivity, thus inhibiting the development of new materials with higher conductivity and/or wider temperature range for operation.

In recent years, hybrid MOF materials have emerged as potential candidates as a new class of proton conductors owing to a number of unique features.^3^–^7^ For example, the functionalisation of organic constituents allows the periodic introduction of acidic groups (*e.g.*, –COOH, –PO_3_H_2_, –SO_3_H), which can facilitate efficient proton transfer pathways.^8^–^14^ However, the synthesis of MOFs bearing free acid groups is very challenging, primarily due to the favoured deprotonation of these acids and their subsequent coordination to the metal nodes under solvothermal conditions. The crystalline nature of MOFs provides an opportunity to understand further the dynamics of proton diffusion, which can be used to inform future materials design. Quasi-elastic neutron scattering (QENS) has been shown recently to be a powerful technique for gaining insights into the mechanisn of proton conduction in crystalline MOFs.^15^,^16^ However, such studies remain very rare to date. Current research into proton conducting MOFs (PCMOFs) can be separated into low temperature (<100 °C) and high temperature (>100 °C) materials.^17^ Low temperature PCMOFs are much more common, and, similar to Nafion, rely on the presence of water to mediate proton transfer through the framework. The hydrogen bonding network of water molecules is usually dictated by the structure and dimensionality of the framework itself and high water-stability is often a critical requirement for PCMOFs.^18^

Herein, we report the progressive design and synthesis of three organic linkers, H_3_L^1^ ([1,1′:3′,1′′-terphenyl]-2′,4,4′′-tricarboxylic acid), H_4_L^2^ ([1,1′:3′,1′′-terphenyl]-4,4′,4′′,6′-tetracarboxylic acid) and H_5_L^3^ ([1,1′:3′,1′′-terphenyl]-2′,4,4′,4′′,6′-pentacarboxylic acid) containing multi-carboxylic acid functionality. The ligands combine with Ba(NO_3_)_2_ to form three MOFs, MFM-510 [Ba_2_(L^1^)(H_2_O)_1.5_(CO_2_)(DMF)_1.5_], MFM-511 [Ba(H_2_L^2^)(H_2_O)(DMF)] and MFM-512 [Ba_2_(HL^3^)(H_2_O)_4_] which are stable to water vapour. MFM-510, in which all carboxylic acids are bound to the metal node, exhibits only moderate proton conductivity (2.1 × 10^–5^ S cm^–1^) at 99% RH and 298 K. Although MFM-511 contains two monodentate carboxylic acid groups which remain protonated, their mobility is highly restricted by strong intramolecular hydrogen bonding to a neighbouring carboxyl oxygen atom, leading to a moderately enhanced conductivity of 5.1 × 10^–5^ S cm^–1^ at 99% RH and 298 K. In comparison, MFM-512, which retains pendant carboxylic acid functionality directed into the unrestricted framework void, shows two orders of magnitude enhancement on proton conductivity (2.9 × 10^–3^ S cm^–1^) under the same conditions. Considering the similar metal–ligand coordination of these three MOFs, these improvements on proton conductivity can be considered as a direct result of the incorporation of free and accessible carboxylic acid groups into MOFs.^13^,^19^,^20^ We also report an investigation of the proton diffusion and dynamics in MFM-512 *via* QENS, which confirms the proton conduction in MFM-512 is mediated by the “free diffusion inside a sphere” mechanism.

## Experimental section

### Materials and characterisations

Starting materials were purchased from Sigma Aldrich and used without further purification. Full experimental details of syntheses and techniques used can be found in the ESI.†

## Results and discussions

### Synthesis and structural characterisation

**Fig. 1:**
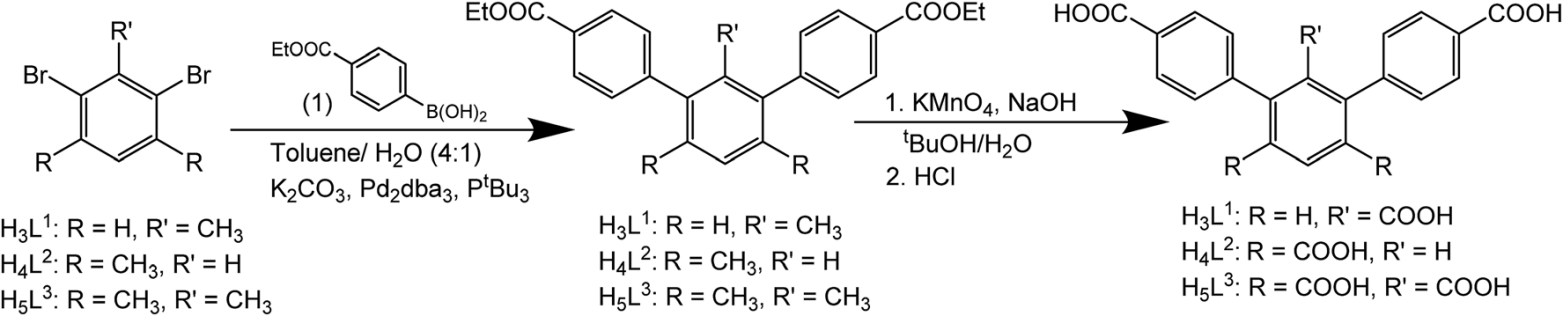
Synthesis of H_3_L^1^, H_4_L^2^ and H_5_L^3^.

H_3_L^1^, H_4_L^2^ and H_5_L^3^ were obtained *via* a two-step synthesis, involving an initial Suzuki–Miyaura cross-coupling reaction between an aryl-dibromo compound with [4-(ethoxycarbonyl)phenyl]boronic acid (Fig. 1). This was followed by oxidation of the methyl groups situated on the central aromatic ring and subsequent hydrolysis of the terminal ester functionalities. MFM-510, -511 and -512 were synthesised under solvothermal conditions by combining the respective ligand with Ba(NO_3_)_2_ and 2 M HCl in a mixture of DMF, ethanol and water before heating in a 5 mL screw top vial at 85 °C for 72 h. All MOFs were obtained as colourless single crystals in a single phase, enabling their structure determination *via* single crystal X-ray diffraction. The synthesis of MFM-510, -511 and -512 can be readily scaled up using an open reaction vessel, and the phase purity of the scaled-up materials has been confirmed by PXRD (Fig. S1–S3†).

**Fig. 2:**
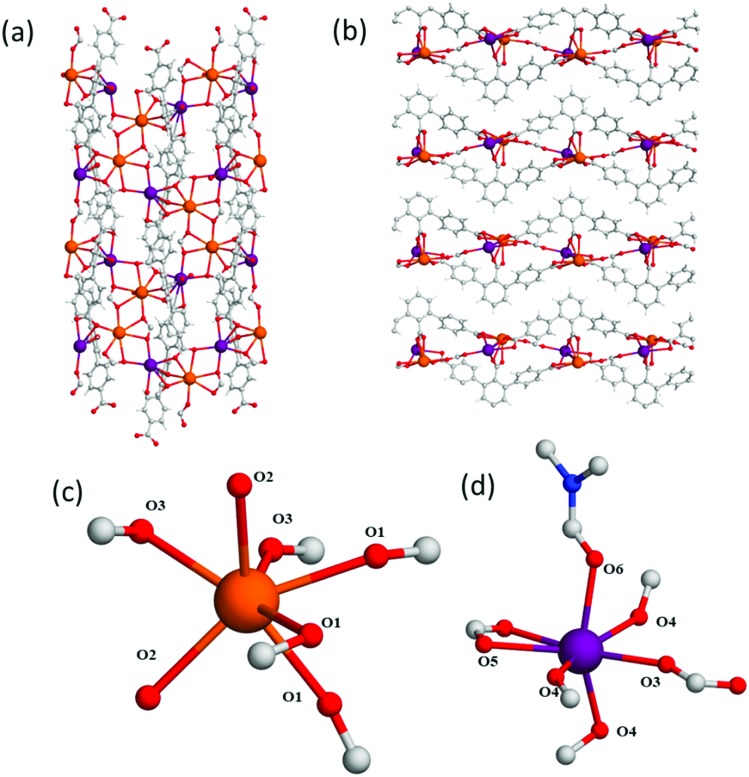
View of the 2D-sheet structure of MFM-510 along (a) the *a*-axis and (b) the *c*-axis. Terminal DMF and water ligands are removed for clarity. View of (c) type I and (d) type II Ba(ii) nodes showing trigonal prismatic coordination. H atoms are omitted for clarity. Carbon, grey; oxygen, red; hydrogen, white; nitrogen, blue; barium, orange (type I) and purple (type II).

MFM-510 crystallises in the *P*2_1_/*c* space group (Table S1†) and contains two types of mononuclear Ba(ii) node with trigonal prismatic coordination (Fig. 2). The type I Ba(ii) center is bound to three monodentate carboxylates (O1), two terminal aquo ligands (O2) and two formate molecules (O3) which bridge to adjacent type II nodes, the formic acid being formed *via* the thermal decomposition of DMF during the synthesis. The type II Ba(ii) nodes are bound by three monodentate carboxylates (O4), one bidentate carboxylate (O5), one bridging formate (O3) and a terminal DMF molecule (O6). The structure of MFM-510 results in H_3_L^1^ being fully deprotonated and bound to Ba(ii) centers by all three carboxylic acid groups, leaving no pendant –COOH groups. The extended framework of MFM-510 is constructed with tightly packed 2D metal–ligand sheets running along the *a*-axis.

**Fig. 3:**
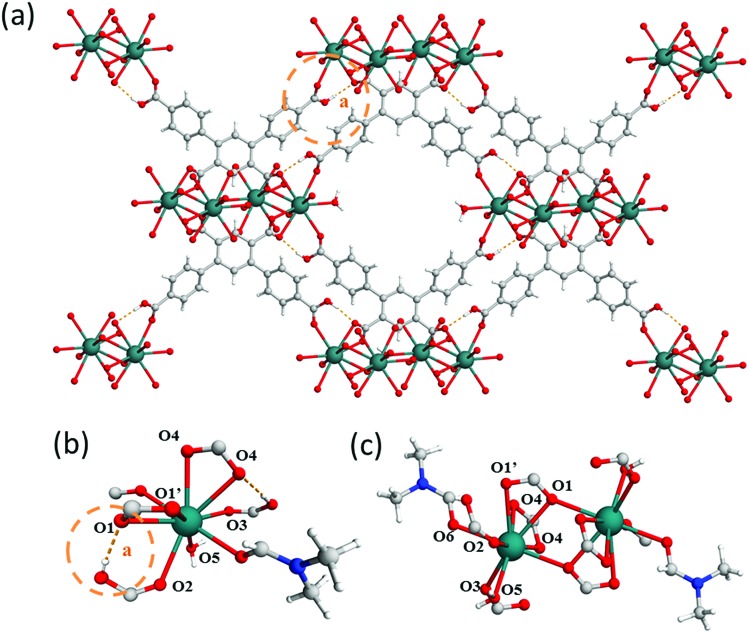
View of (a) the crystal structure in MFM-511 along the *a*-axis, highlighting restrictive hydrogen bonding of carboxyl proton (*a* = 1.79 Å); (b) the Ba(ii) metal node in MFM-511 showing monocapped square antiprismatic coordinate geometry; (c) the binuclear Ba(ii) moiety in MFM-511 viewed along the *a*-axis. Carbon, grey; oxygen, red; hydrogen, white; nitrogen, blue; barium, teal.

The 4-fold COOH-functionalised ligand H_4_L^2^ was targeted to synthesise MFM-511 in an attempt to create pendant carboxylic acid sites. This molecule has two carboxylic acid moieties on its central ring compared to one in H_3_L^1^. It was hypothesised that the steric bulk created by chelation to the highly coordinated Ba(ii) metal centres would lead to at least one of the carboxylic acid groups being retained as a pendant functional group upon formation of a MOF structure. MFM-511 crystallises in the *P*1[combining macron] space group and contains interconnected pores which build to form a 3D open structure (Table S1† and Fig. 3). MFM-511 has one type of Ba(ii) node which consists of a 9-coordinated metal centre with mono-capped square anti-prismatic coordination geometry. Each Ba(ii) is chelated by three monodentate carboxylates (O1, O2, O3), two bidentate carboxylates (O1′, O4), one terminal aquo (O5) and one terminal DMF ligand (O6). Two nodes combine *via* two bridging carboxylates (O1) to form a {Ba_2_O_16_} moiety (Fig. 3c). Eight bi-metallic units {Ba_2_O_16_} connect *via* six linkers to give four small voids which surround a larger central pore (Fig. 3a). The smaller voids (8.7 × 3.5 Å) are decorated by two bound carboxylate groups. The large central pore (14 × 12 Å) is decorated by terminal water molecules which are 10 Å apart for the O···O separation. Unfortunately, as with MFM-510, all of the carboxyl groups in MFM-511 are coordinated to Ba(ii) centres with no free pendant carboxylic acid groups present. However, two monodentate carboxyl O atoms adjacent to O2 and O3 remain protonated, but their accessibility is severely hindered as they point into the smaller void of the framework. The mobility of these carboxyl protons is also highly restricted due to a very strong intramolecular hydrogen bonding interaction with neighbouring O atoms (O···O = 1.79(1) Å; Fig. 3a and b). As a result, there is an absence of accessible free carboxylic acid groups in the structure of MFM-511, potentially limiting the proton conductivity of this material.

**Fig. 4:**
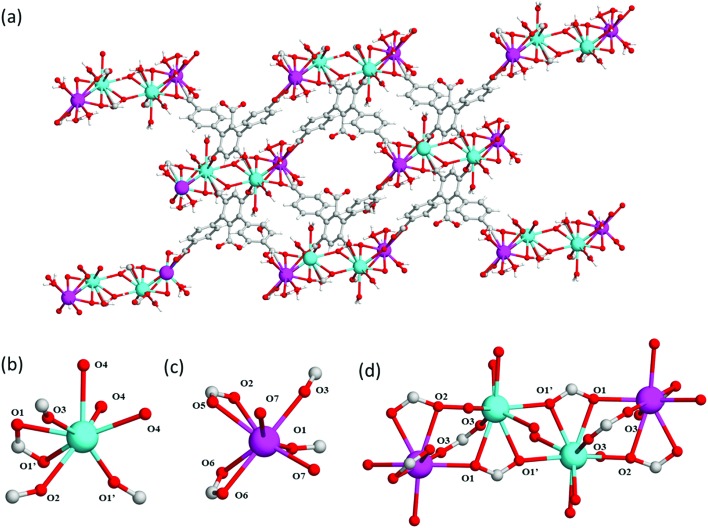
View of (a) the crystal structure of MFM-512 along the *a*-axis; (b) type (I) and (c) type II Ba(ii) nodes in MFM-512 showing distorted octahedral and trigonal prismatic square face monocapped coordination, respectively. H-atoms are omitted for clarity; (d) View of the tetranuclear Ba(ii) cluster in MFM-512. Carbon, grey; oxygen, red; hydrogen, white; barium, magenta (type I) and cyan (type II).

In a further attempt to generate pendant –COOH groups within a MOF, a third ligand, H_5_L^3^, was targeted to form MFM-512 *via* reaction with Ba(ii) cations. MFM-512 crystallises in the *P*1[combining macron] space group and exhibits, as planned, pendant carboxylic acid groups in the structure (Table S1,† Fig. 4a). It contains two types of mononuclear Ba(ii) nodes which show dodecahedral and square anti-prismatic coordination geometries. The type I nodes are chelated by one bidentate carboxylate (O1, O1′), three monodentate carboxylates (O1′, O2, O3) and three terminal aquo ligands (O4) (Fig. 4b). The type II nodes are chelated by two bidentate carboxylates (O2, O5, O6), two monodentate carboxylates (O1, O3) and two terminal aquo ligands (O7) (Fig. 4c). Type I and type II nodes combine to form tetra-metallic clusters, {Ba_4_O_27_} in which two-central type I metal nodes are bridged by two carboxylates (O1′) and terminal type II nodes are bridged to the central type I nodes by a further three carboxylates (O1, O2, O3) (Fig. 4d). In MFM-512, the central ring of each ligand connects to the two-central type I Ba(ii) centers within the metal cluster and the terminal rings of the ligand bridge a further two clusters through their terminal, type II Ba(ii) nodes (Fig. 4a).

**Fig. 5:**
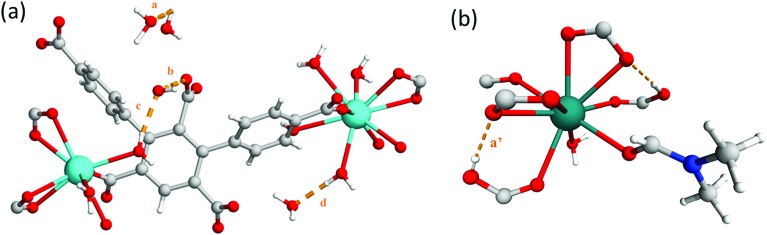
View of (a) the crystal structure of MFM-512 showing hypothetical hydrogen bonding network with unbound water present in pores, *a* = 2.51 Å, *b* = 1.93 Å, *c* = 2.05 Å, *d* = 1.88 Å. (b) View of Ba(ii) node in MFM-511 showing restriction of the carboxyl proton *a*′ = 1.79 Å.

The framework of MFM-512 contains interconnected pores which build to form a similar 3D open structure to MFM-511. In this case, the connection of four {Ba_4_O_27_} clusters *via* six organic linkers results in four small voids (6.5 × 8.2 Å) which are decorated by terminal aquo ligands and surround a larger central void (8.6 × 10.4 Å) (smaller than that of MFM-511) which hosts two pendant carboxylic acid groups. The distance between free carboxylic acids (O···O) is 4.8083(2) Å, the shortest distance between terminal aquo ligands (O···O) is 4.3782(2) Å, and the shortest distance between free carboxylic acid and terminal aquo ligands (O···O) is 5.1649(3) Å. These distances are longer than those reported for PCMOFs,^15^,^21^–^23^ which show an intrinsic hydrogen bonding network. For example, in MFM-500(M) (M = Ni, Co) the bond distances between uncoordinated phosphonate hydroxyl groups and coordinated aquo ligands is between 2.00 and 2.90 Å.^15^ Despite the proton donor–acceptor distances in the MFM-512 framework being too far apart for them to form an intrinsic hydrogen bonding network, the single crystal structure suggests that the presence of unbound water molecules in the central pore may facilitate a hydrogen bonding network involving the pendant carboxylic acid groups, with hypothetical hydrogen bond distances in the range of 1.93–2.51 Å. The hydrogen bonding interactions in MFM-512 are considerably weaker than those in MFM-511 meaning that the mobility of the carboxyl protons is less restricted (Fig. 5). This increases the possibility of an effective hydrogen bonding network being formed for proton conductivity in the presence of free water.

### Studies of the proton conductivity

The proton conductivity of MFM-510, -511, and -512 was evaluated using AC impedance analysis on a pelletised sample of each MOF at 298 K at variable humidity (Table 1 and Fig. S13–S18†). For each of the Nyquist plots obtained, the often incomplete semi-circle in the high-frequency region represents the bulk and grain boundary resistances, while the low frequency tail represents the blocking of protons at the electrode interface, indicating ionic conduction. The proton conductivity of all three MOFs showed significant dependence on humidity with the maximum conductivity obtained at 99% RH, consistent with many literature PCMOFs.^24^–^31^ Despite the lack of pendant carboxylic acid groups in both MFM-510 and MFM-511, the retention of protonated carboxyl groups coupled with the terminal aqua ligands in MFM-511 lead to an approximate two-fold increase in proton conductivity compared to MFM-510. The maximum conductivity of MFM-510 and MFM-511 is 2.1 × 10^–5^ and 5.1 × 10^–5^ S cm^–1^, respectively, which is moderate compared to low temperature PCMOFs found in the literature (Table S3†). The low conductivity of MFM-510 and MFM-511 is attributed to the lack of accessible, mobile protons within the framework structure. Under the same conditions, MFM-512 exhibits a significantly improved (100-fold) proton conductivity of 2.9 × 10^–3^ S cm^–1^. This suggests that the availability of accessible mobile protons within the MOF from the pendant carboxylic acid moiety contributes directly to improving proton conductivity. Other examples within the literature have shown a similar trend for MOFs decorated with various protic functional groups^10^,^13^,^14^ and, interestingly, MFM-512 is amongst the highest conducting PCMOFs analysed under similar conditions (Table S3†). More importantly, in comparison to other MOFs which rely on pendant carboxylic acid functionality specifically,^13^,^19^ MFM-512 shows the highest conductivity at 99% RH and room temperature. Despite MFM-511 having an analogous structure to MFM-512 and containing protonated carboxyl groups, its proton conductivity is considerably lower at 5.1 × 10^–5^ S cm^–1^, once again suggesting the accessibility (*i.e.*, unrestricted motions) of pendant carboxylic acid groups plays a key role in maximising and enhancing conduction properties in these materials.

**Table 1:** Summary of the proton conductivity at 298 K and variable humidity for MFM-510, -511 and -512

MFM-510		MFM-511		MFM-512	
% RH	*σ* (S cm^–1^)	% RH	*σ* (S cm^–1^)	% RH	*σ* (S cm^–1^)
99	2.1 × 10^–5^	99	5.1 × 10^–5^	99	2.9 × 10^–3^
90	1.1 × 10^–6^	93	2.4 × 10^–7^	95	1.5 × 10^–4^
80	2.4 × 10^–7^	87	4.0 × 10^–8^	77	2.5 × 10^–7^
60	<10^–9^	51	< 10^–9^	59	<10^–9^

To probe the activation energies for proton conductivity, variable temperature impedance measurements were conducted at 95% RH to achieve stable humidity over a wide range of temperatures. The activation energies are 0.40 and 0.32 eV for MFM-511 and MFM-512, respectively, suggesting a Grotthuss proton transport mechanism (Fig. S20†). MFM-510 has an activation energy of 0.63 eV, which is more indicative of a vehicle proton transport mechanism. This suggests that proton diffusion is less facile in MFM-510 compared to the other two MOFs, consistent with its reduced proton conductivity.

### Studies of water sorption

**Fig. 6:**
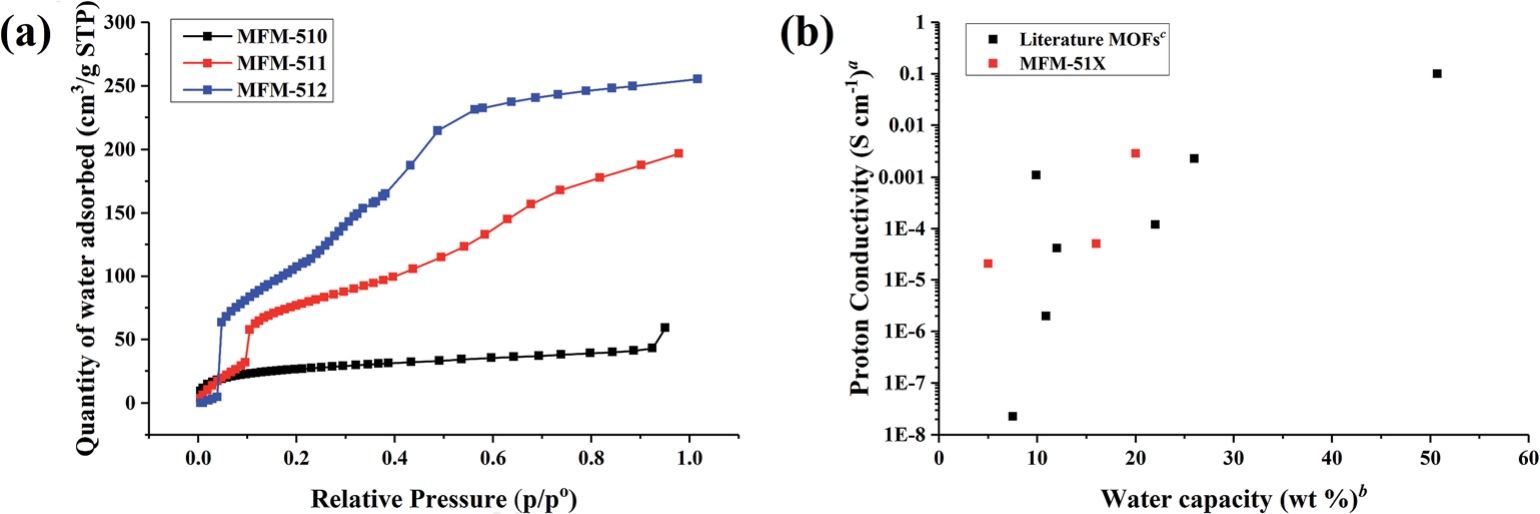
(a) Adsorption isotherms for water in MFM-510, -511, and -512. Desorption isotherms are shown in ESI† for clarity. (b) Relationship between water capacity and proton conductivity for MFM-510, -511, and -512 and a selection of MOFs in literature. ^*a*^Impedance measurements at high humidity and room temperature, full details in Table S4.† ^*b*^Water capacity at the RH of the impedance data. ^*c*^Water uptake assessed by volumetric or gravimetric analysis.

In order to obtain a deeper understanding of the dependence of proton conductivity on humidity in these MOFs, water sorption isotherms were measured at 298 K for all three MOFs (Fig. 6a). MFM-510 shows a very low uptake of water regardless of humidity, suggesting that the closely packed 2D sheets of MFM-510 limit the amount of water able to enter the MOF, in good agreement with its poor proton conductivity. MFM-511 and MFM-512 both show three-step water uptake profiles, with an initial low uptake at low humidity followed by a two-step pore filling process. The first pore filling steps of MFM-511 and MFM-512 starts at 10 and 5% RH, respectively, highlighting the higher hydrophilicity of MFM-512 likely due to hydrogen bonding from guest water to the accessible pendant –COOH groups in the larger pores of MFM-512. This is in contrast to the highly restricted protonated carboxyl groups located in the smaller pores of MFM-511. The water capacity of MFM-510, -511 and -512 at ∼90% RH was 59, 197 and 255 cm^3^ g^–1^ (5, 16, 20 wt%), respectively, which is comparable to the literature.^9^,^19^,^25^,^26^,^28^,^31^–^39^ Measurement of water sorption above 90% RH is challenging due to the condensation of water on the external surface of MOF crystallites. Interestingly, the trend in water adsorption shows excellent agreement with the conductivity at 99% RH and 298 K in these MOFs (Fig. 6b). The hysteresis present in desorption cycles in all cases indicates the presence of hydrophilic channels in these MOFs (Fig. S21–S23†). All three MOFs show negligible N_2_ sorption uptake (Fig. S24–S26†) consistent with the larger kinetic diameter of N_2_ compared to water, the uptake of water being enhanced further by internal hydrogen bonding interactions within the MOF.

### QENS study of proton diffusion in MFM-512

To gain an understanding of the mechanism of proton dynamics in MFM-512, QENS spectra of MFM-512 were recorded between 248 and 423 K. In a QENS experiment, diffusional dynamics are probed by studying changes in inelastic scattering near the elastic signal at zero energy transfer. Such processes manifest themselves in QENS spectra as a decrease in the elastic intensity corresponding to coherent scattering, accompanied by a broadening of the linewidth associated with incoherent scattering of individual atoms. As the neutron coherent scattering cross-section is notably larger for hydrogen compared to other elements, the observed broadening can be assigned mainly to hydrogen atoms from the MOF. The crystal structure of MFM-512 suggests that the mobile hydrogen atoms are from water molecules bound to the metal and from the pendant carboxylic acids, as the hydrogen atoms on the phenyl rings are tightly bound.

**Fig. 7:**
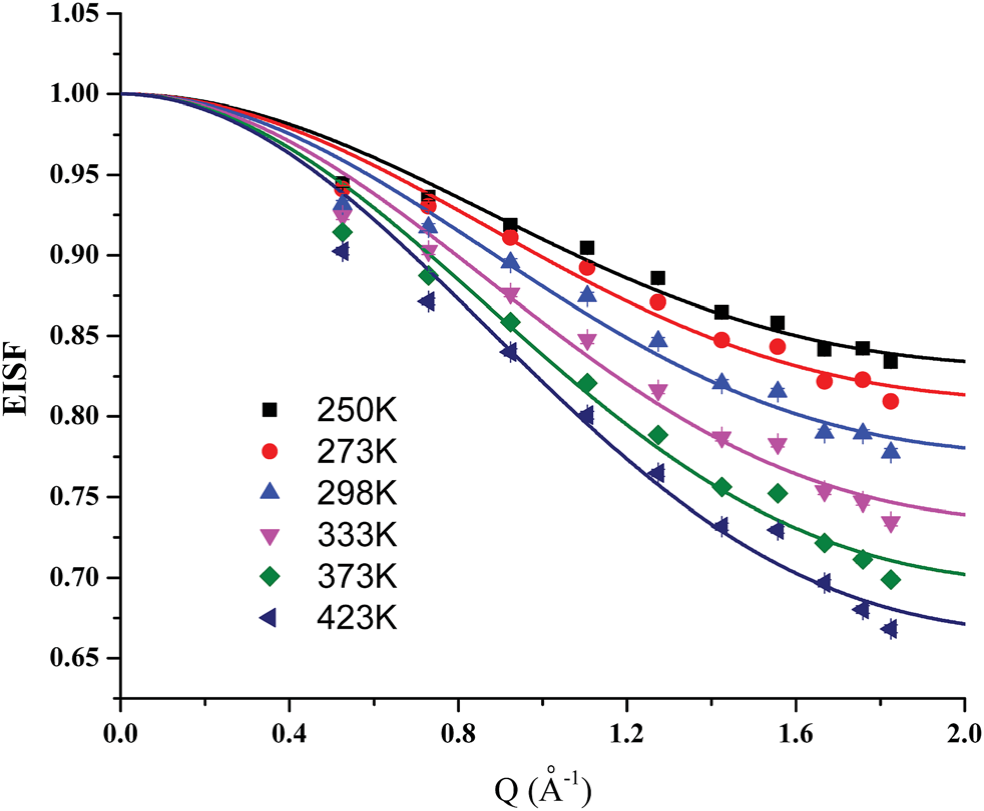
Plots of elastic incoherent structure factor (EISF) for MFM-512. Solid curves represent the simulated EISF (eqn (1)) using the model of “free diffusion inside a sphere” at the corresponding temperatures.

The elastic incoherent structure factor (EISF) measures the contribution of the elastic scattering to the total scattering, with different diffusive modes possessing different *Q*-dependence (where *Q* is momentum transfer). It is thus possible to investigate the geometrical information of the free protons in MFM-512 by calculating the EISFs. For MFM-512, the EISF plots show obvious *Q*-dependence (Fig. 7) and can be successfully fitted using the “free diffusion inside a sphere” model (eqn (1)).^15^

1 EISF = *p* + (1 – *p*)[3*j*_1_(*Qr*)/(*Qr*)]^2^

where *j*_1_ is the first-order spherical *Bessel* function, *r* is the radius of the sphere, and *p* and (1 – *p*) are the immobile and mobile fraction involved in the diffusion process, respectively. The fitting resulted in *r* = 1.8995 Å (Table S5†) which is in excellent agreement with the approximate hydrogen bond distances observed crystallographically for MFM-512 (Fig. 5a). This emphasises the need for an effective hydrogen bonding network within the framework structure which becomes increasingly viable as increasing amounts of water are adsorbed under high humidity conditions. Overall, this result is consistent with the activation energy measurement from impedance spectroscopy data that suggested a Grotthuss ‘proton-hopping’ mechanism, which necessitates short distances between adjacent sites.

It is worth noting that whilst both NMR methods and QENS probe diffusional dynamics within molecular systems, they are complementary techniques in that different time- and space-scales are probed. QENS focusses on shorter time lengths at the atomic scale. The complementary nature of such techniques has been highlighted when looking at hydrated Nafion membranes, where QENS data have been used to probe in more depth the dynamics of water, with highly consistent diffusion coefficients as those obtained from the macromolecular-scale pulsed-field gradient NMR studies.^40^ In another example, both pulse-field gradient NMR and QENS experiments have been used to study the diffusion of ammonia in silicalite, and it was found that the different timescales of the experiments permitted different diffusive processes to be probed.^41^

## Conclusions

Three new water-vapour stable MOFs, MFM-510, -511, -512, have been synthesised from a family of organic ligands containing multiple carboxylic acid groups. It is worth noting that these three MOFs are not iso-structural and materials conductivity can stem from a combination of many factors. In this work, we focus primarily on the study of the role of free carboxylic acid moiety on proton conductivity. MFM-512 is the only example that incorporates accessible free carboxylic acid moiety within the structure. In MFM-510 the carboxylates are completely bound to the metal centres and despite the presence of protonated carboxyl groups in MFM-511, these are highly restricted and hindered for proton diffusion. Proton conductivity studies confirm that accessible free carboxylic acid groups within the framework leads to a 100-fold enhancement in conductivity in MFM-512. MFM-512 is highly competitive with other low temperature PCMOFs reported in the literature under similar conditions. An investigation of proton dynamics in MFM-512 by QENS confirms clear mediation of proton conduction by “free diffusion inside a sphere” mechanism.

## Conflicts of interest

The authors declare no competing financial interests.

## Supplementary Material

Supplementary information

Crystal structure data
