# Supplementary material for: Modulating proton diffusion and conductivity in metal–organic frameworks by incorporation of accessible free carboxylic acid groups
Source: Chem Sci. 2018 Nov 2;10(5):1492–9. doi: 10.1039/c8sc03022g (PMC6354967; doi:10.1039/c8sc03022g)
Supplement: Supplementary file 1 [file SC-010-C8SC03022G-s001.pdf]

Electronic Supplementary Material (ESI) for Chemical Science.
This journal is © The Royal Society of Chemistry 2018

Supporting Information

Modulating proton diffusion and conductivity in metal-organic frameworks by incorporation of accessible free carboxylic acid groups

Peter Rought,[1#] Christopher Marsh,[1#] Simona Pili,[1] Ian P. Silverwood,[2] Victoria García Sakai,[2] Ming Li,[3] Martyn S. Brown,[1] Stephen P. Argent,[4] Inigo Vitorica-Yrezabal,[1] George Whitehead,[1] Mark R. Warren,[5] Sihai Yang[1]* and Martin Schröder[1]*

1. School of Chemistry, University of Manchester, Manchester M13 9PL, U.K.
   Sihai.Yang@manchester.ac.uk; M.Schroder@manchester.ac.uk
2. ISIS Pulsed Neutron and Muon Source, Rutherford Appleton Laboratory, Oxfordshire OX11 0QX, U.K.
3. School of Engineering, University of Nottingham, Nottingham NG7 2RD, U.K.
4. School of Chemistry, University of Warwick, Coventry CV4 7AL, U.K.
5. Diamond Light Source, Harwell Science and Innovation Campus, Oxfordshire OX11 0DE, U.K.

[#]: these authors contribute equally to this work.

1. Methods

General experimental characterisation

^{1}H NMR spectra were recorded using either a B400 Bruker Avance III 400 MHz or B500 Bruker Avance II+ 500 MHz. Chemical shifts are reported in parts per million relative to tetramethylsilane. Mass spectrometry of samples were performed in MeOH or MeCN using a Waters SQD2 (Q-MS with ES+, ES- and APCI source) spectrometer. The ATR-IR spectra were collected on a Thermo Scientific Nicolet iS5-IR spectrometer in the 4000-400 cm^{-1} range. Elemental analyses were performed on a Flash 2000 elemental analyser. X-ray powder diffraction patterns were collected on a PANalytical MPD powder diffractometer using a plate sample holder at room temperature. N_2 sorption and BET measurements were made using a Micromeritics Tristar porosity analyser at 77 K. Water vapour sorption isotherms were measured using a Micromeritics 3Flex sorption instrument at 293 K. Thermogravimetric analyses, differential scanning calorimetry and mass spectrometry (TGA-DSC-MS) were recorded on a TA instruments Q600 TGA attached to a Hiden Analytical DSMS under air with a flow rate of 100 mL/min and a heating rate of 5 ^oC/min.

Single crystal X-ray diffraction

X-ray data for MFM-510 were collected at 120 K using a GV1000 Oxford-Rigaku Supernova diffractometer with Cu-Kα (λ = 1.54178Å) equipped with a CCD detector and an Oxford Cryosystems N_2 flow system. Data were measured using CrysAlisPro suit of programs. X-ray data for compound MFM-511 were collected at 150 K using an Oxford-Rigaku Supernova diffractometer with Mo-Kα (λ = 0.71073Å) equipped with a CCD detector and an Oxford Cryosystems N_2 flow system. Data were measured using CrysAlisPro suit of programs. X-Ray data for compound MFM-512 were collected at a temperature of 120 K using a synchrotron radiation at single crystal X-ray diffraction beamline I19 in Diamond light Source,[S1] equipped with an Pilatus 2M detector and an Oxford Cryosystems N_2 flow system. Data were measured using GDA suite of programs.

Crystal structure determination and refinement

X-Ray data for MFM-510 and MFM-511 were processed and reduced using CrysAlisPro suite of programmes. Absorption correction was performed using empirical methods (SCALE3 ABSPACK) based upon symmetry-equivalent reflections combined with measurements at different azimuthal angles.[S2] X-ray data for MFM-512 were processed and reduced using dials suite of programmes.[S3] Absorption correction was performed using empirical methods (SADABS). The crystal structures were solved and refined against all F^2 values using SHELXL and Olex 2 suite of programmes.[S4] All atoms were refined anisotropically with hydrogen atoms placed in calculated positions. DMF solvent molecules and some water molecules were heavily disordered and modelled over two positions. The C-N, and C-O distances in the DMF were restrained using DFIX and SADI commands. The atomic displacement parameters (ADP) have been restrained using RIGU and SIMU commands. A number of A and B alerts were found due to absorption correction problems in compounds MFM-510 and MFM-512. CCDC 1849703-1849705 contains the supplementary crystallographic data for this paper. These data can be obtained free of charge *via* www.ccdc.cam.ac.uk/conts/retrieving.html (or from the

Cambridge Crystallographic Data Centre, 12 Union Road, Cambridge CB21EZ, UK; fax: (+44)1223-336-033; or deposit@ccdc.cam.ac.uk).

Proton conductivity measurements

Proton conductivity measurements were performed on a Solartron SI1260 Impedance analyser over a frequency range of 0.1 Hz to 1 MHz at an amplitude of 100 mV and 0 mV DC rest voltage. Conductivity measurements were carried out on pressed pellets of finely ground powder samples. Samples for conductivity measurements were prepared by grinding the sample (~0.1 g) into a homogeneous powder with a mortar and pestle, added to a standard 8 mm die, coated with a conductive silver paste to improve contact with the two blocking electrodes, and pressed at 5,000 kg for 5 minutes. Resultant pellets of 8 mm in diameter and a thickness of ~1.05 mm were placed in the electrochemical cell. Relative humidity of 99% was obtained using a Kambic KK-50 climatic chamber. The proton conductivity (σ, S cm^{-1}) was calculated from the impedance data, using the following equation: $\sigma = l/RS$, where l and S are the thickness (cm) and cross-sectional area (cm^2) of the pellet respectively, and R, which was extracted from the impedance plots, is the total resistance of the sample (Ω). ZView software was used to analyse the impedance data.

2. Syntheses

Synthesis of diethyl 2'-methyl-[1,1':3',1''-terphenyl]-4,4''-dicarboxylate.

2,6-Dibromotoluene (1.9 g, 7.6 mmol), **1** (3.55 g, 18 mmol) and K_2CO_3 (4.04 g, 29 mmol) were added to a mixture of toluene (200 mL) and H_2O (50 mL). The resulting solution was heated to 60 °C and degassed for 15 mins under an atmosphere of N_2. A 1 M solution of $P(^tBu)_3$ in toluene (3.4 mL, 3.22 mmol) and Pd_2dba_3 (1.06 g, 1.14 mmol) were added to the vigorously stirred reaction mixture which was then heated to 80 °C under N_2. Reaction completion was confirmed by TLC (1:3 EtOAc/hexane) against starting materials after 6 h. The resulting solution was filtered, H_2O was added to the filtrate and the crude product was extracted with CH_2Cl_2. The organic phase was dried over $MgSO_4$ and evaporated under reduced pressure. The resulting solid was recrystallised from hot MeOH to afford the desired product (2.36 g, 80%). ^1H NMR (500 MHz, $CDCl_3$) δ = 8.10 (d, 4H, ArH, J = 7.5 Hz), 7.43 (d, 4H, ArH, J = 7.5 Hz), 7.32 (t, 1H, ArH, J = 7.5 Hz), 7.24 (d, 2H, J = 7.5 Hz), 4.40 (q, 4H, CH_2, J = 7.5 Hz), 2.07 (s, 3H, CH_3), 1.41 (t, 6H, CH_3, J = 7.5 Hz). ^{13}C NMR ($CDCl_3$, 125 MHz): 166.52, 146.80, 142.60, 135.99, 129.43, 129.36, 129.22, 129.10, 125.63, 61.00, 18.58, 14.36. MS (ESI) [m/z] = 389.2 $[M+H]^+$.

Synthesis of [1,1':3',1''-terphenyl]-2',4,4''-tricarboxylic acid (H_3L^1).

Diethyl 2'-methyl-[1,1':3',1''-terphenyl]-4,4''-dicarboxylate (1.00 g, 2.6 mmol) and NaOH (2.89 g, 72 mmol) were stirred in ^tBuOH (50 mL) and H_2O (100 mL). The solution was heated to 50 °C and $KMnO_4$ (8.14 g, 52 mmol) was added gradually over 2 days until the reaction solution remained purple. On addition of 2/3 of $KMnO_4$, the temperature was increased to 70 °C. Once the addition was complete, ^iPrOH (50 mL) was added and the reaction mixture heated at reflux for 2 h. The resulting suspension was filtered and washed with boiling H_2O. The filtrate was concentrated to 50 mL and acidified to pH 1 with 12 M HCl (5 mL). The product was

isolated by filtration to yield a white solid (0.851 g, 91%). ^{1}H NMR (CD_3OD, 400 MHz) δ = 8.09 (d, 4H, ArH, *J* = 8.4 Hz), 7.63 (t, 1H, ArH, *J* = 7.8 Hz) 7.58 (d, 4H, ArH, *J* = 8.4 Hz), 7.49 (d, 2H, ArH, 7.8 Hz). ^{13}C NMR (CD_3OD, 125 MHz): 169.71, 146.71, 140.51, 135.24, 131.31, 130.88, 130.71, 130.58, 129.98. MS(ESI) [m/z] = 385.1 $[M+Na]^+$. FTIR-ATR ν = 2394, 1697, 1610, 1411, 1232, 764.

Synthesis of $[Ba_2(L^1)(H_2O)_2(CO_2)(DMF)]$, (MFM-510).

$Ba(NO_3)_2$ (25 mg, 0.096 mmol) and H_3L^1 (10 mg, 0.03 mmol) were dissolved in DMF (2 mL), H_2O (0.5 mL), EtOH (0.3 mL) and 2 M HCl (0.05 mL) in a 5 mL wheaton vial. The mixture was heated at 85 °C for 72 h. The resulting colourless needles were washed with boiling DMF:H_2O:EtOH (1:1:1) then acetone to yield MFM-510. Elemental Analysis (%): found: C 36.83, H 2.89, N 2.09. Calculated C 38.15 H 2.82 N 1.78. FTIR-ATR ν = 2820, 1681, 1530, 1411, 1341, 1276, 731.

Synthesis of (4-(ethoxycarbonyl)phenyl)boronic acid (1).

4-Boronobenzoic acid (20.00 g, 103 mmol) was added to a mixture of conc. H_2SO_4 and EtOH (500 mL). The reaction mixture was refluxed for 12 h. The resulting solution was filtered to remove any impurities and the filtrate concentrated to ca. 100 mL. A precipitate was obtained on addition of H_2O and was collected by filtration, washed with excess H_2O to pH 7 and dried under vacuum to afford the desired white product (13.26 g, 78%). ^{1}H NMR (270 MHz, dmso-D_6) δ = 8.05 (d, 2H, *J* = 5.4 Hz), 7.80 (d, 2H, *J* = 5.4 Hz), 4.38 (q, 2H, *J* = 7.0 Hz), 1.39 (d, 3H, *J* = 7.0 Hz). MS (ESI) [m/z] 194.9 $[M+H]^+$.

Synthesis of diethyl 4',6'-dimethyl-[1,1':3',1''-terphenyl]-4,4''-dicarboxylate (3).

1,5-Dibromo-2,4-dimethylbenzene (2.53 g, 9.5 mmol), **1** (4.47 g, 23 mmol) and K_2CO_3 (6.64 g, 48 mmol) were added to a mixture of toluene (200 mL) and H_2O (50 mL). The resulting solution was heated to 60 °C and degassed for 15 mins under N_2. A 1 M solution of $P(^tBu)_3$ in toluene (3.4 mL, 3.22 mmol) and Pd_2dba_3 (1.06 g, 1.14 mmol) were added to the vigorously stirred reaction mixture which was then heated to 80 °C under N_2. Reaction completion was confirmed after 6 h by TLC (1:3 EtOAc/hexane) against starting materials. The resulting solution was filtered, H_2O was added to the filtrate and the crude product was extracted with CH_2Cl_2. The organic phase was dried over $MgSO_4$ and solvent evaporated under reduced pressure. The resulting solid was recrystallised from hot MeOH to afford the desired product (2.04 g, 53%). ^{1}H NMR (500 MHz, $CDCl_3$) δ = 8.09 (d, 4H, ArH *J* = 7.4 Hz), 7.43 (d, 4H, ArH, *J* = 7.4 Hz), 7.23 (s, 1H, ArH), 7.12 (s, 1H, ArH), 4.40 (q, 4H, *J* = 7.8 Hz), 2.30 (s, 6H), 1.41 (t, 6H, *J* = 7.8 Hz). ^{13}C NMR ($CDCl_3$, 100 MHz): 166.57, 146.05, 138.66, 134.77, 132.79, 130.69, 129.43, 129.29, 128.98, 61.00, 20.01, 14.38. MS (ESI) [m/z] = 403.2 $[M+H]^+$.

[1,1':3',1''-terphenyl]-4,4',4'',6'-tetracarboxylic acid (H_4L^2).

3 (1.02 g, 2.54 mmol) and NaOH (2.69 g, 67 mmol) were stirred in ^{t}BuOH (50 mL) and H_2O (100 mL). The solution was heated to 50 °C and $KMnO_4$ (10.62 g, 67 mmol) was added gradually over 2 days until the reaction solution remained purple. On addition of 2/3 of $KMnO_4$, the temperature was increased to 70 °C. Once the addition was complete, ^{i}PrOH (50 mL) was added and the reaction mixture heated at reflux for 2 h. The

resulting suspension was filtered and washed with boiling H_2O. The filtrate was concentrated to 50 mL and acidified to pH 1 with 12 M HCl (5 mL). The product was isolated by filtration to yield a white solid (0.8 g, 70%). ^{1}H NMR (CD_3OD, 400 MHz): 8.41 (s, 1H, ArH), 8.07 (d, 4H, ArH, *J* = 8.4 Hz), 7.51 (d, 4H, ArH, *J* = 8.4 Hz), 7.42 (s, 1H, ArH). ^{13}C NMR (DMSO-*d*$_6$), 100 MHz): 168.04, 167.15, 144.14, 142.94, 132.85, 131.06, 130.02, 129.12, 128.76. MS (ESI) [m/z] = 405.2 $[M-H]^-$ FTIR-ATR ν = 3157, 2451, 1682. 1607, 1573, 1545, 1282, 707.

Synthesis of [Ba(H_2L^2)(DMF)(H_2O)], (MFM-511).

$Ba(NO_3)_2$ (250 mg, 1 mmol) and H_4L^2 (110 mg, 0.27 mmol) were dissolved in DMF (20 mL), H_2O (5 mL), EtOH (3 mL) and 2 M HCl (0.5 mL). The mixture was heated at 85 ^{o}C with stirring for 72 h. The resulting white crystalline solid was obtained by filtration and washed with boiling DMF:H_2O:EtOH (1:1:1) then acetone to yield MFM-511 (0.10 g, 59%). Elemental Analysis (%): found: C 47.89, H 3.12, N 2.37. Calculated C 47.60 H 3.04 N 2.22. FTIR-ATR ν = 2935, 2514, 1680, 1665, 1651, 1610, 1603, 1588, 1531, 1260, 693.

Synthesis of diethyl 2',4',6'-trimethyl-[1,1':3',1''-terphenyl]-4,4''-dicarboxylate (4).

Dibromomesitylene (2.66 g, 9.5 mmol), **1** (4.47 g, 23 mmol) and K_2CO_3 (6.64 g, 48 mmol) were added to a mixture of toluene (200 mL) and H_2O (50 mL). The resulting solution was heated to 60 ^{o}C and degassed for 15 minutes under an atmosphere of N_2. A 1 M solution of P(^{t}Bu)$_3$ in toluene (3.4 mL, 3.22 mmol) and Pd_2dba_3 (1.06 g, 1.14 mmol) were added to the vigorously stirred reaction mixture which was then heated to 80 ^{o}C under N_2. Reaction completion was confirmed after 6 h by TLC (1:3 EtOAc/hexane) against start materials. The resulting solution was filtered, H_2O was added to the filtrate and the crude product was extracted with DCM. The organic phase was dried over $MgSO_4$ and evaporated under reduced pressure. The resulting solid was recrystallised from hot MeOH to afford the desired product (3.94 g, 100%). ^{1}H NMR (400 MHz, $CDCl_3$) δ = 8.13 (d, 4H, ArH *J* = 8.4 Hz), 7.27 (d, 4H, ArH, *J* = 8.4 Hz), 7.08 (s, 1H, ArH), 4.41 (q, 4H, *J* = 7.2 Hz), 2.03 (s, 6H), 1.66 (s, 3H), 1.42 (t, 6H, *J* = 7.2 Hz). ^{13}C NMR ($CDCl_3$, 125 MHz): 188.93, 166.58, 143.32, 134.77, 130.49, 128.95, 128.37, 125.40, 60.94, 50.87 20.71, 18.87, 14.35**.** MS (ESI) [m/z] = 417.2 $[M+H]^+$.

Synthesis of [1,1':3',1''-terphenyl]-2',4,4',4'',6'-pentacarboxylic acid (H_5L^3).

4 (1.40 g, 3.4 mmol) and NaOH (2.69 g, 67 mmol) were stirred in ^{t}BuOH (50 mL) and H_2O (100 mL). The solution was heated to 50 ^{o}C and $KMnO_4$ (10.62 g, 67 mmol) was added gradually over 2 days until the reaction solution remained purple. On addition of 2/3 of $KMnO_4$, the temperature was increased to 70 ^{o}C. Once the addition was complete, ^{i}PrOH (50 mL) was added and the reaction mixture heated at reflux for 2 h. The resulting suspension was filtered and washed with boiling H_2O. The filtrate was concentrated to 50 mL and acidified to pH 1 with 12 M HCl (5 mL). The product was isolated by filtration to yield a white solid (0.771 g, 51%). ^{1}H NMR (CD_3OD, 500 MHz): 8.47 (s, 1H, ArH), 8.01 (d, 4H, ArH, *J* = 7.5 Hz), 7.42 (d, 4H, ArH, *J* = 7.5 Hz). ^{13}C NMR (DMSO-d_6, 100 MHz): 168.47, 167.58, 144.58, 143.37, 133.28, 131.49, 130.45, 129.56, 129.20. MS (ESI) [m/z] = 451.1 $[M+H]^+$. FTIR-ATR ν = 2428, 1714, 1668, 1562, 1534, 1252, 1233, 736.

Synthesis of [$Ba_2(HL^3)(H_2O)_4$], (MFM-512).

$Ba(NO_3)_2$ (500 mg, 2 mmol) and H_5L^3 (240 mg, 0.5 mmol) were dissolved in DMF (40 mL), H_2O (10 mL), EtOH (6 mL) and 2 M HCl (1 mL). The mixture was heated at 85 ^oC with stirring for 72 h. The resulting colourless needles were obtained by filtration and washed with boiling DMF:H_2O:EtOH (1:1:1) then acetone to yield MFM-512 (0.163 g, 39%). Elemental Analysis (%): found: C 34.32, H 2.44, N 0.00. Calculated C 34.83 H 2.29 N 0. FTIR-ATR ν = 3331, 1722, 1676, 1562, 1534, 1252, 1233, 697.

3. Single crystal X-ray structures

Table S1: Single crystal X-ray diffraction parameters for MFM-510, -511, -512

Identification code	MFM-510	MFM-511	MFM-512
Empirical formula	$C_{26.5}H_{25.5}Ba_2N_{1.5}O_{11}$	$C_{25}H_{21}BaNO_{10}$	$C_{24.5}H_{31.5}Ba_2N_{0.5}O_{19.5}$
Formula weight	815.66	632.77	919.68
Temperature/K	120	150	120
Crystal system	monoclinic	triclinic	Triclinic
Space group	$P2_1/c$	P-1	P-1
a/Å	11.44352(15)	7.9225(3)	9.3111(3)
b/Å	17.2735(2)	12.5631(6)	12.4288(4)
c/Å	14.1871(2)	14.0398(5)	14.3649(4)
α/°	90	114.960(4)	83.592(2)
β/°	92.3020(13)	94.483(3)	85.579(2)
γ/°	90	104.346(4)	82.416(2)
Volume/Å^3	2802.08(7)	1200.53(9)	1634.26(6)
Z	4	2	2
ρ_{calc}g/cm^3	1.933	1.750	1.869
μ/mm^{-1}	22.270	1.715	2.296
F(000)	1580.0	628.0	900.0
Crystal size/mm^3	0.64 × 0.207 × 0.089	0.55 × 0.34 × 0.12	0.6 × 0.05 × 0.05
Radiation	Cu-Kα (λ = 1.54184)	Mo-Kα (λ = 0.71073)	Mo-Kα (λ = 0.6889)
2Θ range for data collection/°	7.732 to 146.928	6.546 to 58.684	5.674 to 54.996
Index ranges	-14 ≤ h ≤ 13, -21 ≤ k ≤ 15, -17 ≤ l ≤ 17	-10 ≤ h ≤ 9, -17 ≤ k ≤ 16, -18 ≤ l ≤ 17	-12 ≤ h ≤ 12, -16 ≤ k ≤ 16, -19 ≤ l ≤ 19
Reflections collected	15093	9848	24057
Independent reflections	5518 [R_{int} = 0.0580, R_{sigma} = 0.0537]	5491 [R_{int} = 0.0382, R_{sigma} = 0.0508]	8090 [R_{int} = 0.0930, R_{sigma} = 0.0897]
Data/restraints/parameters	5518/377/410	5491/4/338	8090/68/471
Goodness-of-fit on F^2	1.066	1.057	1.124
Final R indexes [I>=2σ (I)]	R_1 = 0.0440, wR_2 = 0.1151	R_1 = 0.0336, wR_2 = 0.0832	R_1 = 0.0529, wR_2 = 0.1540
Final R indexes [all data]	R_1 = 0.0455, wR_2 = 0.1169	R_1 = 0.0374, wR_2 = 0.0865	R_1 = 0.0626, wR_2 = 0.1605
Largest diff. peak/hole /eÅ^{-3}	1.44/-2.63	1.09/-0.91	2.53/-1.67
CCDC	1849703	1849705	1849704

4. Powder X-ray diffraction (PXRD)

PXRD was used to confirm the stability of all three MOFs to impedance measurements at 298 K and 99% RH (99% RH) (Figures S1-S3).

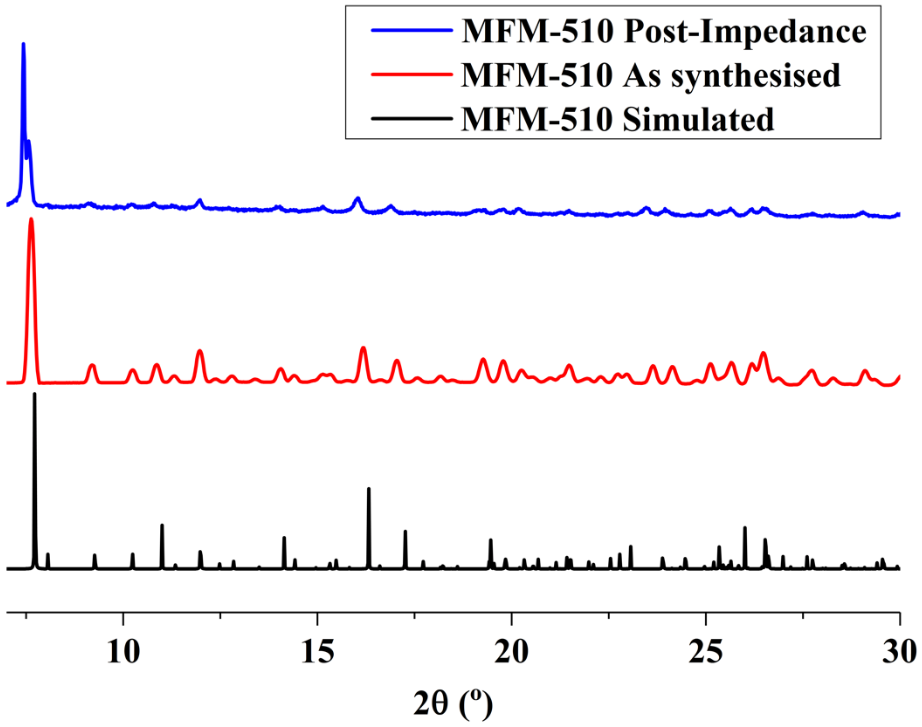

Figure S1: PXRD patterns for MFM-510. Black = simulated from single crystal data, red = as synthesised, blue = post-impedance measurement.

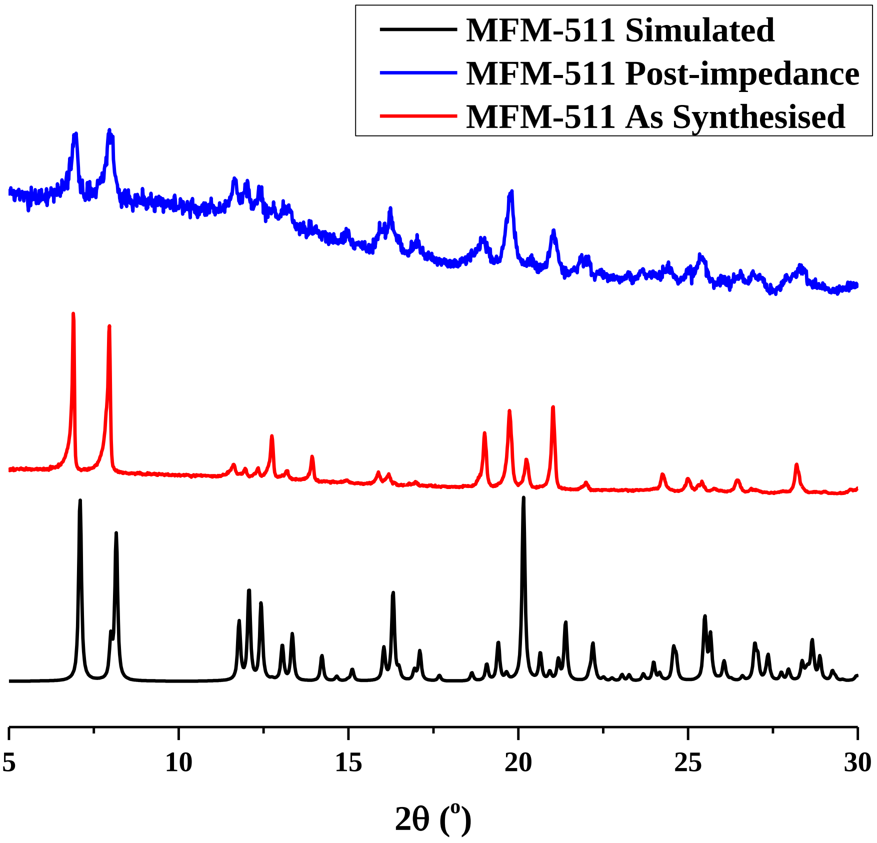

Figure S2: PXRD patterns for MFM-511. Black = simulated from single crystal data, red = as synthesised, blue = post-impedance measurement.

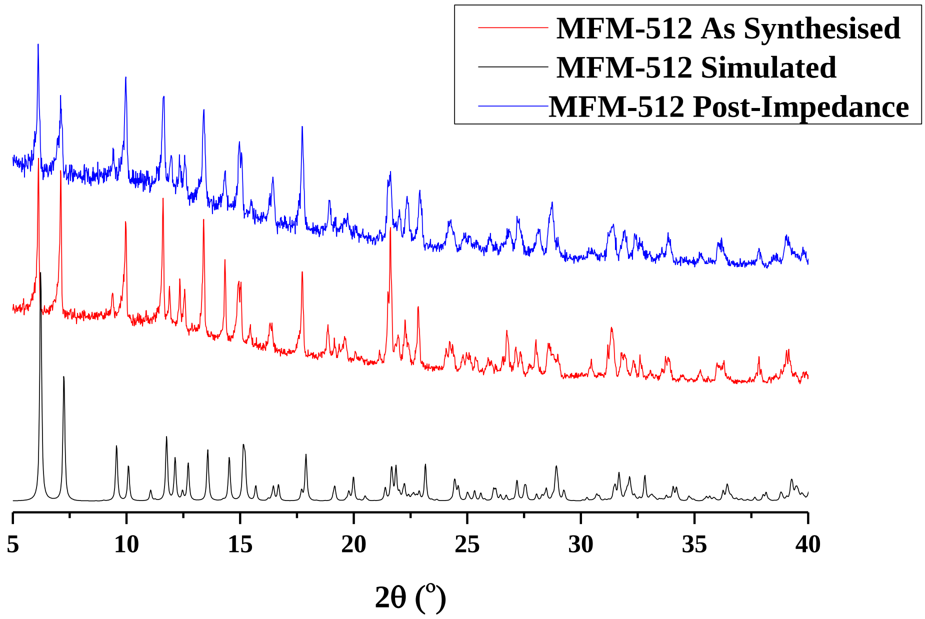

Figure S3: PXRD patterns for MFM-512. Black = simulated from single crystal data, red = as synthesised, blue = post-impedance measurement.

5. Infrared spectroscopy

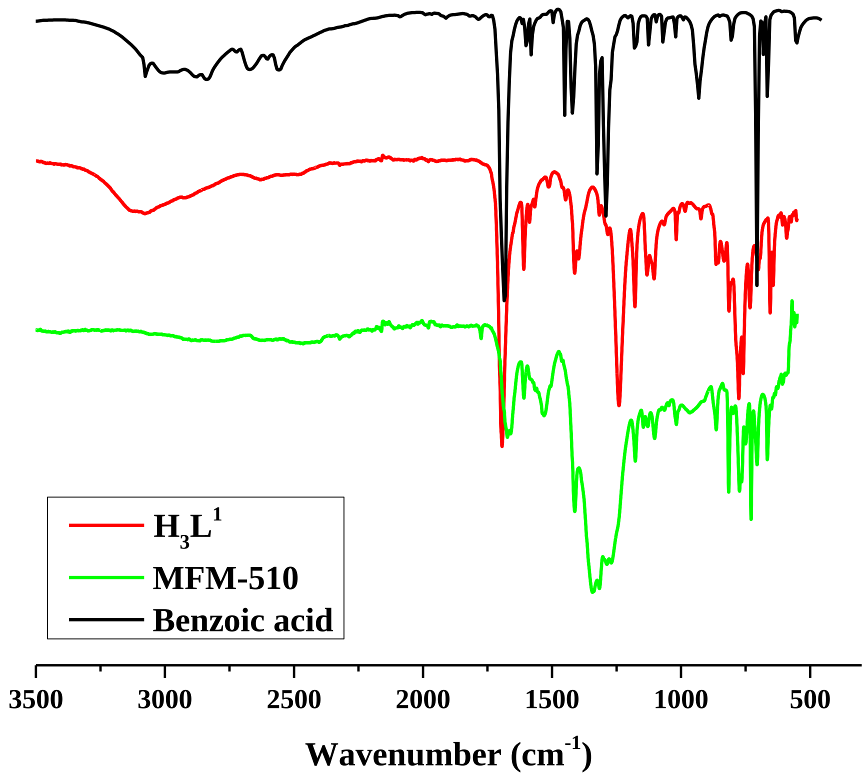

Figure S4: ATR-IR spectra for H_3L^1 (red) and MFM-510 (green). Benzoic acid (black) was used for peak assignment.

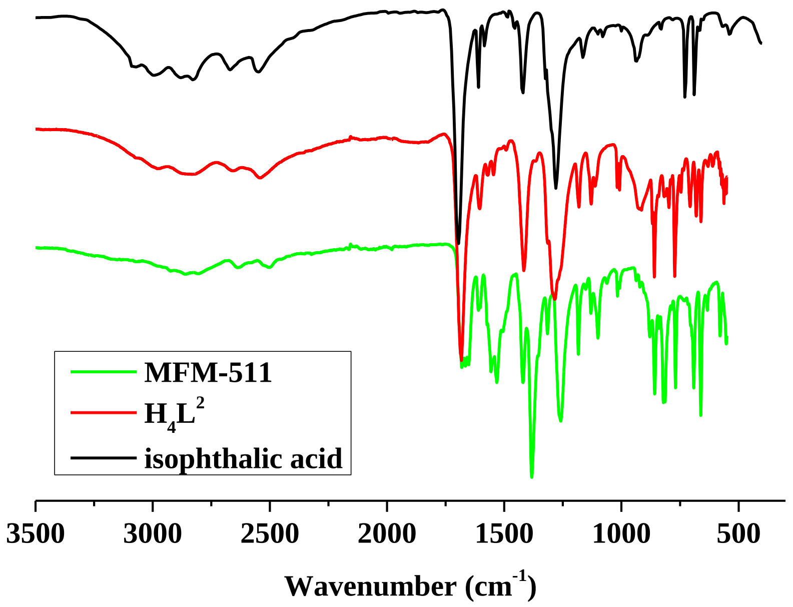

Figure S5: ATR-IR spectra for H_4L^2 (blue) and MFM-511 (green). Benzoic acid (black) and isophthalic acid (red) were used for peak assignment.

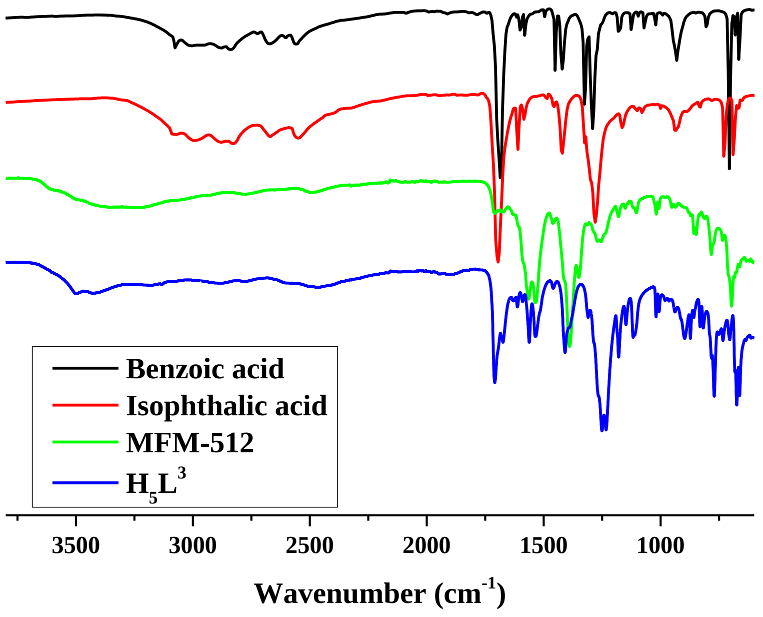

Figure S6: ATR-IR spectra for H_5L^3 (red) and MFM-512 (green). Benzoic acid (black) was used for peak assignment.

6. TGA-DSC-MS

Thermal stability

TGA-DSC-MS data for MFM-510, -511 and -512 were collected between 25 and 600 °C with a heating rate of 5 °C min^{-1} under a flow of air (Figures S7-S12). In all materials, the mass spectometric signal for DMF and acetone, the solvents used during the synthesis of the MOFs is at the baseline level indicating their absence or very low level. For MFM-510, despite being non-porous an initial mass loss (~10%) is observed between 30 and 200 °C, possibly due to evaporation of weakly bound solvent on the surface of the material. A second mass loss between 280 and 350 °C (~10%) was assigned to bound water within the framework and was associated with an endothermic event in the DSC plot. The final mass loss at 425 °C was assigned to framework decomposition. For MFM-511, the initial mass loss (~10%) at around 100 °C was assigned to evaporation of unbound solvent, characterised as water in the MS. A second mass loss (~10%) at around 375 °C is due to bound water within the framework. A final sharp mass loss above 400 °C is attributed to decomposition of the framework. The relatively high decomposition temperature of MFM-510, -511 and -512 offers an advantage for use in fuel cell applications. For MFM-512, the initial mass loss (~10%) between 51 and 165 °C was assigned to evaporation of unbound solvent, characterised as water and a relatively small amount of acetone (which was introduced during the isolation of samples) in the MS. A second mass loss (~15%) between 365 and 423 °C is due to bound water within the framework structure and is accompanied by an endothermic process. A final sharp mass loss (~20%) between 440 and 600 °C is attributed to decomposition of the framework.

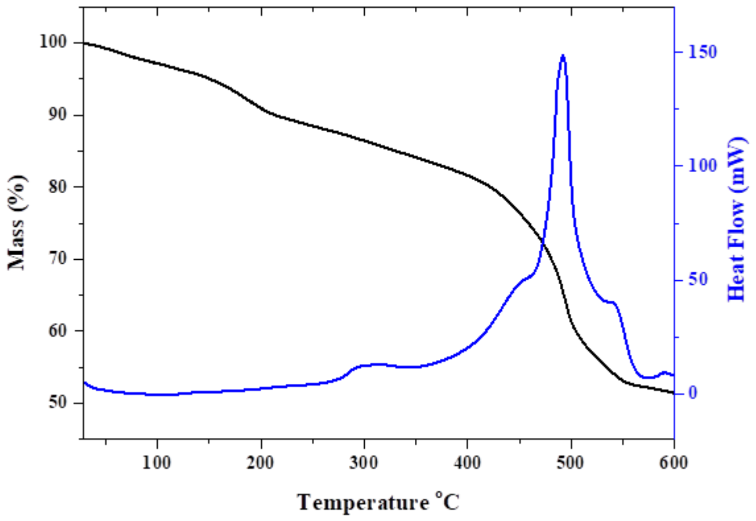

Figure S7: TGA-DSC trace for MFM-510.

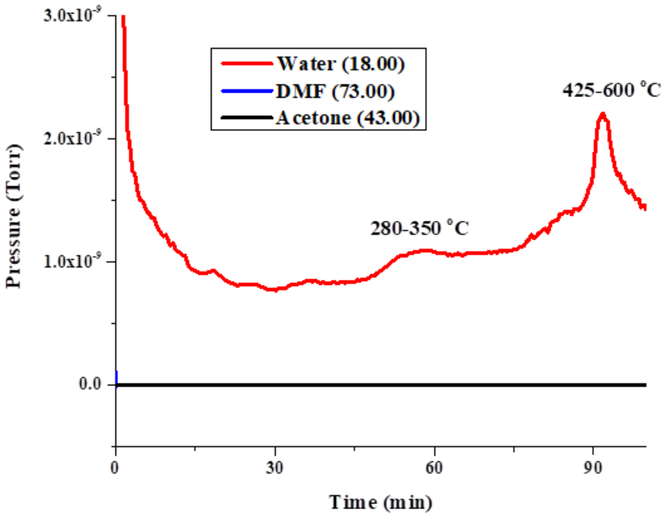

Figure S8: MS trace from TGA eluent for water, DMF and acetone for MFM-510.

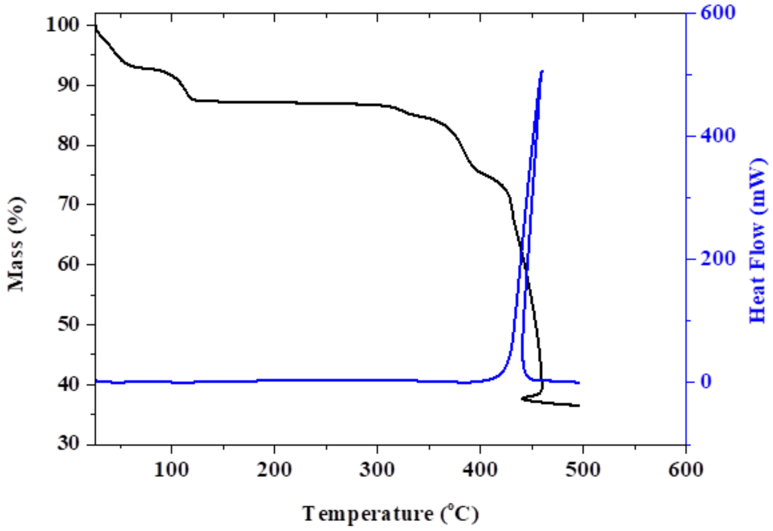

Figure S9: TGA-DSC trace for MFM-511.

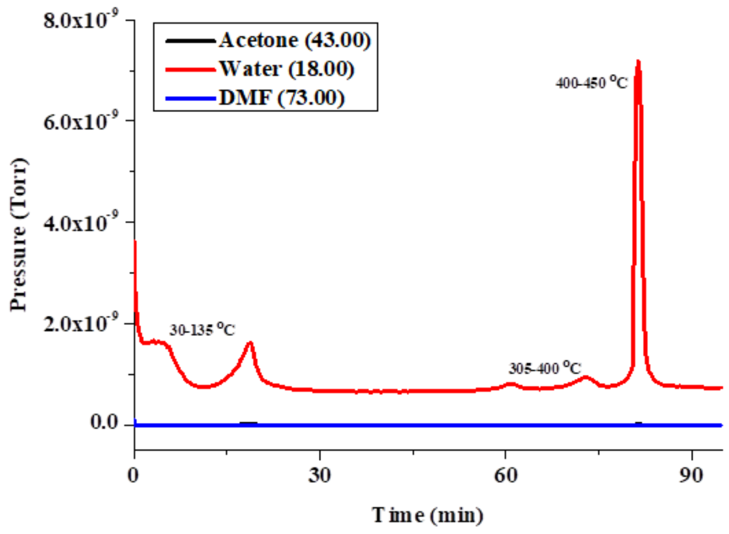

Figure S10: MS trace from TGA eluent for water, DMF and acetone for MFM-511.

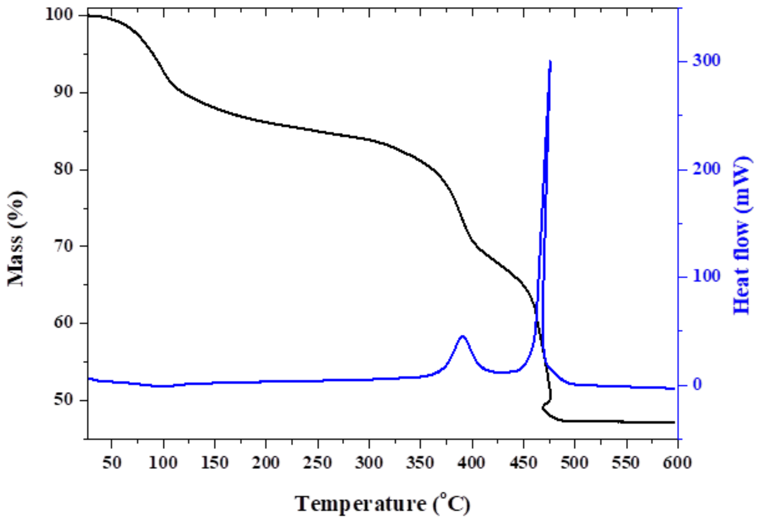

Figure S11: TGA-DSC trace for MFM-512.

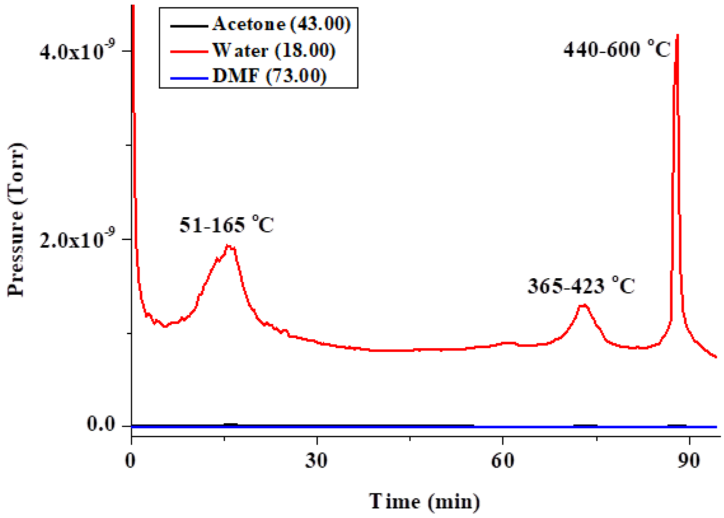

Figure S12: MS trace from TGA eluent for water, DMF and acetone for MFM-512.

7. Proton conductivity

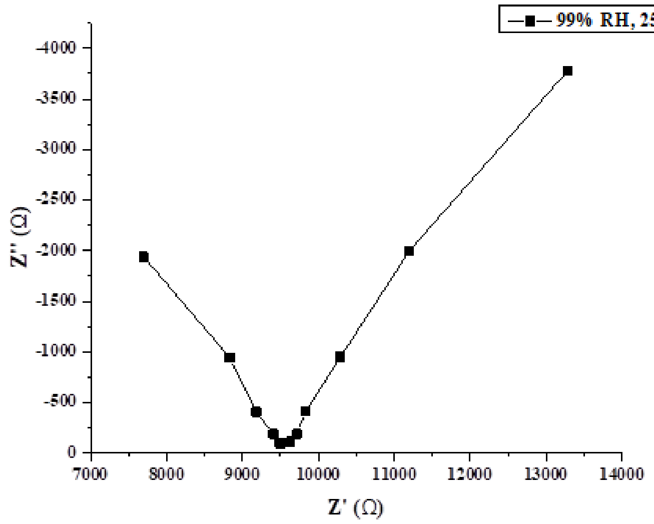

Figure S13: Nyquist plot for MFM-510 recorded at 99% RH and 298 K.

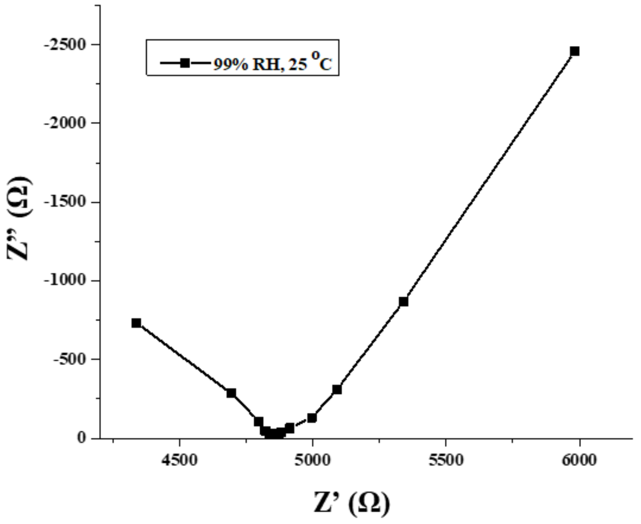

Figure S14: Nyquist plot for MFM-511 recorded at 99% RH and 298 K.

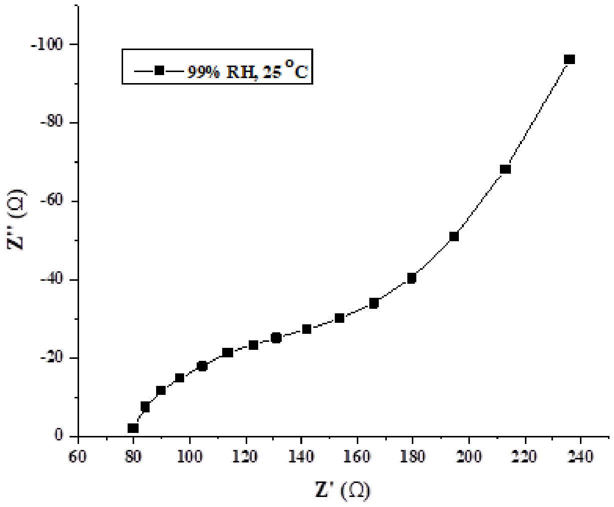

Figure S15: Nyquist plot for MFM-512 recorded at 99% RH and 298 K.

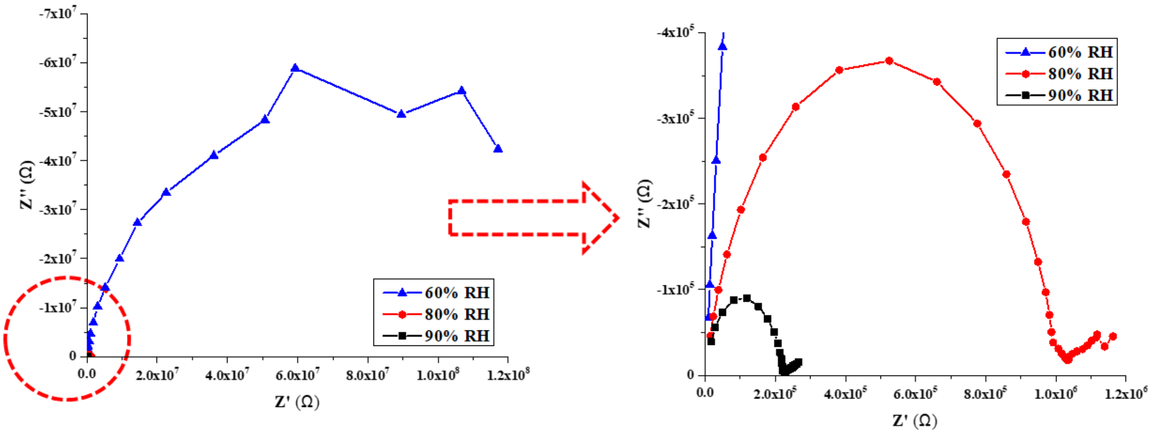

Figure S16: Nyquist plots for MFM-510 recorded at variable humidity and 298 K.

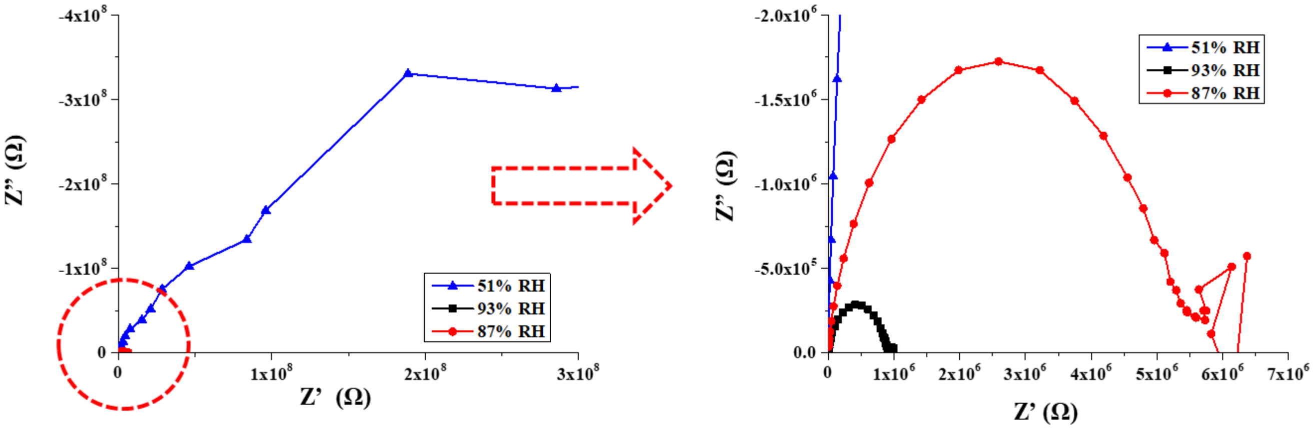

Figure S17: Nyquist plots for MFM-511 recorded at variable humidity and 298 K.

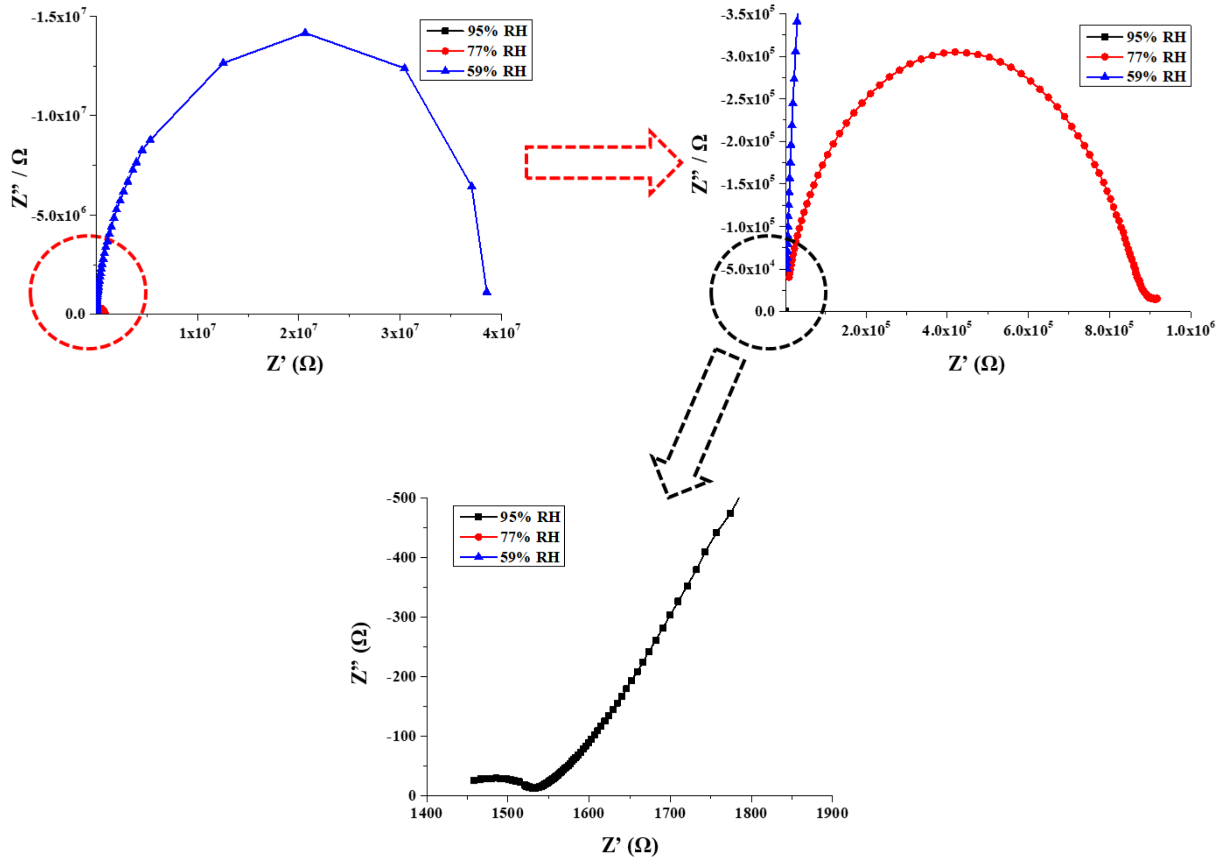

Figure S18: Nyquist plots for MFM-512 recorded at variable humidity and 298 K.

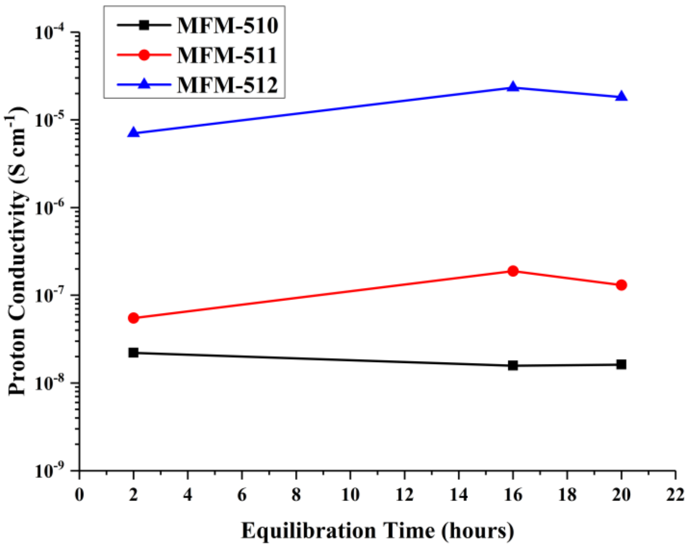

Figure S19: Repeated proton conductivity data of MFM-510, -511, -512 over 1 day at 90% RH, 298 K, demonstrating repeatability and stability of measurements.

Table S2: Summary of proton conductivity data for three separate samples of each MOF, showing reproducibility at 99% RH, 298 K

	**Proton Conductivity at 99% RH, 298 K (S cm^{-1})**		
	**MFM-510**	**MFM-511**	**MFM-512**
Sample 1	2.10×10^{-5}	2.07×10^{-5}	2.89×10^{-3}
Sample 2	2.38×10^{-5}	5.10×10^{-5}	2.09×10^{-3}
Sample 3	6.03×10^{-6}	1.74×10^{-5}	2.63×10^{-3}

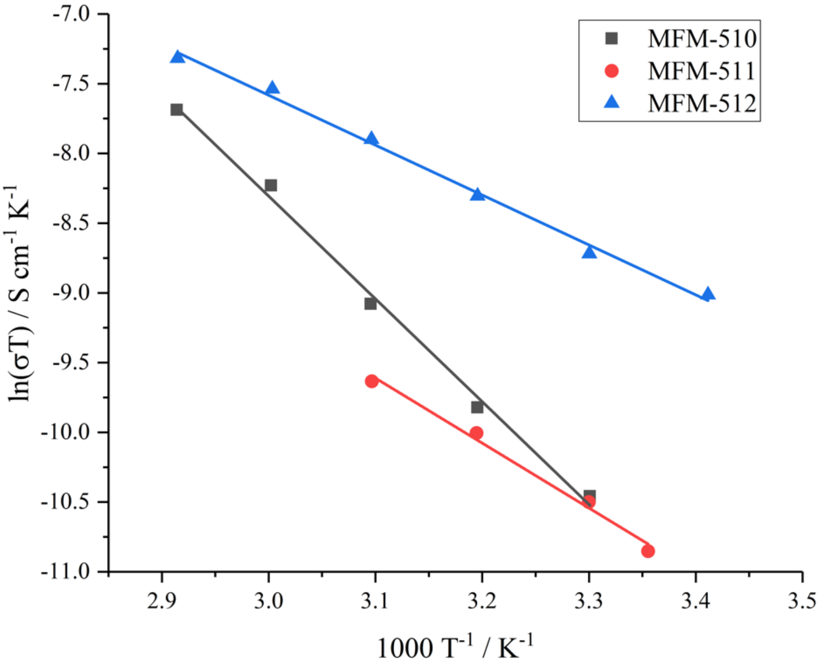

Figure S20: Arrhenius plots of MFM-510, -511 and -512 used for calculation of activation energies at 90% RH

8. Literature comparisons

Table S3: Summary of the proton conductivity of a selection of the best performing low temperature PCMOFs.

MOFs*	σ (S cm^{-1})	T (K)	RH (%)	Ref.
$[(Me_2NH_2)_3(SO_4)]_2[Zn_2(ox)_3]$	4.2×10^{-2}	298	98	5
$La(H_5DTMP)\cdot 7H_2O$	8×10^{-3}	297	98	6
Ca-PiPhtA-NH_3	6.6×10^{-3}	297	98	7
Cu−TCPP nanosheet	3.9×10^{-3}	298	98	8
In-IA-2D-1	3.4×10^{-3}	297	98	9
UiO-66-SO_3H	3.4 x 10^{-3}	303	97	10
UiO-66-2COOH	1.0 x 10^{-3}	303	97	
$Fe(ox)\cdot 2H_2O$	1.3 x 10^{-3}	298	>99	11

*H_8DTMP = hexamethylenediamine-N,N,N′,N′-tetrakis(methylenephosphonic acid), H_2PiPhtA = 5-(dihydroxyphosphoryl)isophthalic acid, H_4TCPP = Tetrakis(4-carboxyphenyl)porphyrin, H_2IA = Isophthalic acid, ox^{2-} = oxalate.

9. Additional sorption isotherms

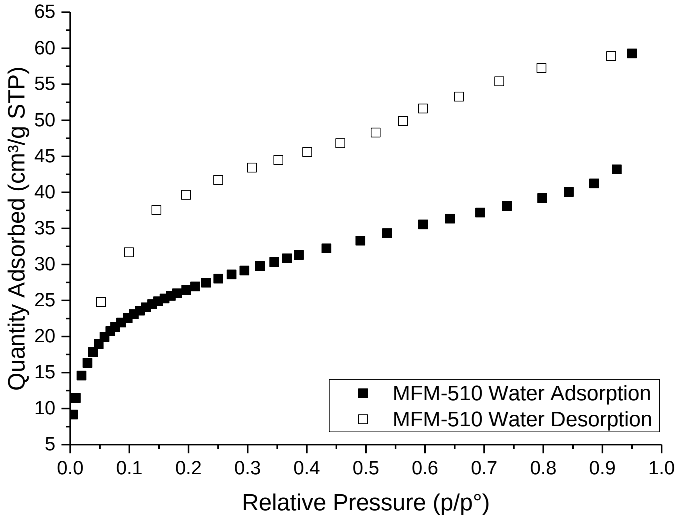

Figure S21: Water vapour sorption isotherm for MFM-510 recorded at 298 K.

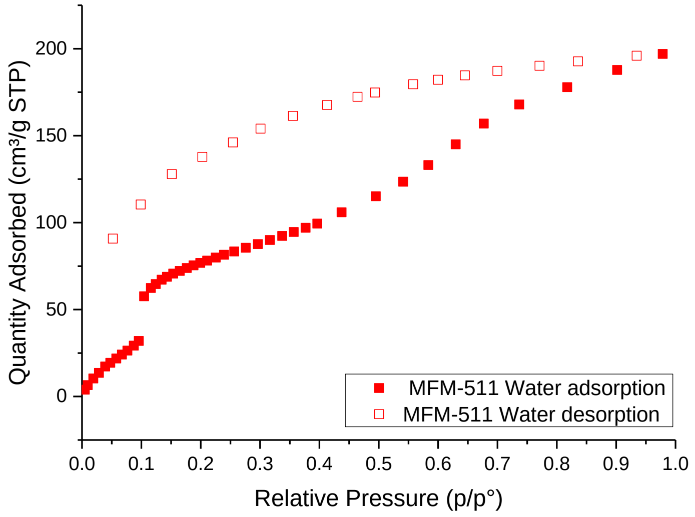

Figure S22: Water vapour sorption isotherm for MFM-511 recorded at 298 K.

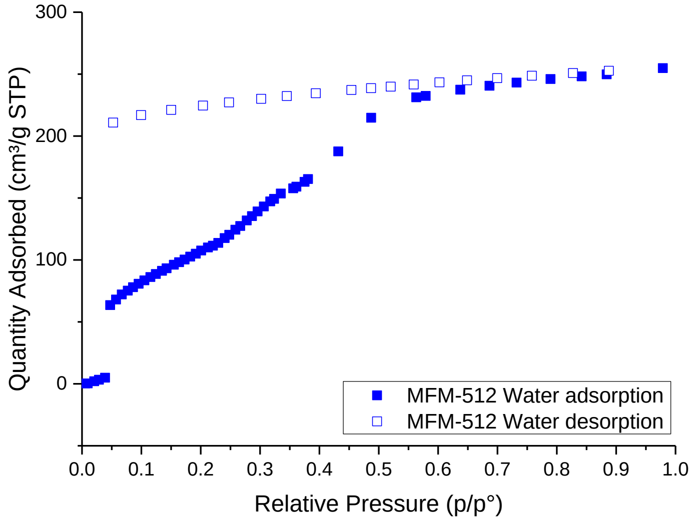

Figure S23: Water vapour sorption isotherm for MFM-512 recorded at 298 K.

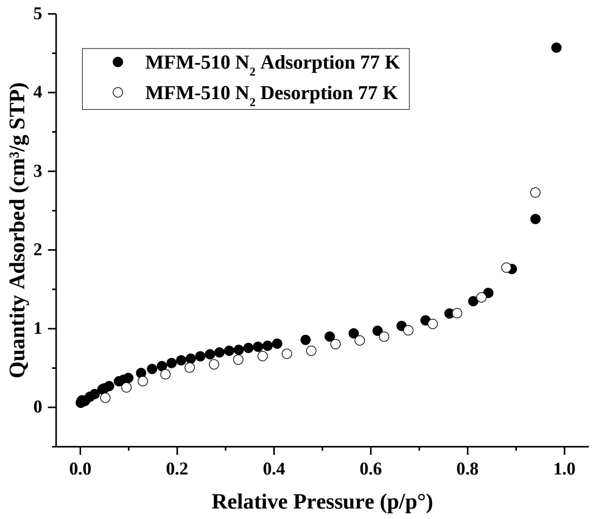

Figure S24: N_2 sorption isotherm for MFM-510 recorded at 77 K. BET surface area = 2.69(4) m^2/g.

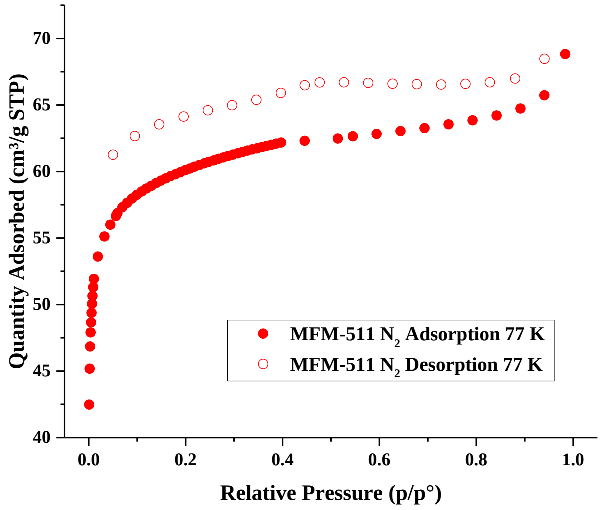

Figure S25: N_2 sorption isotherm for MFM-511 recorded at 77 K. BET surface area = 185(3) m²/g

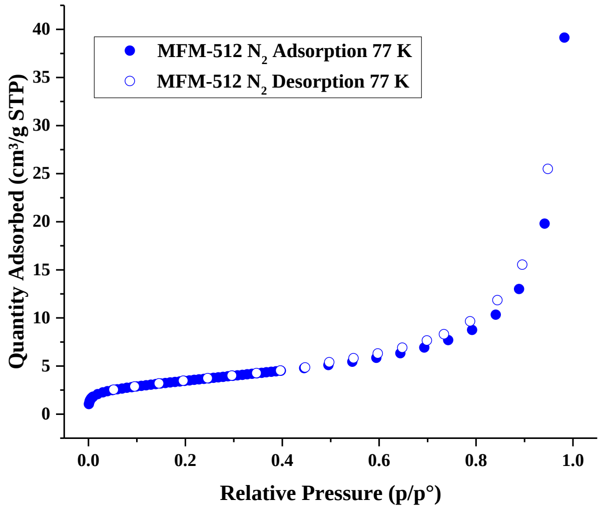

Figure S26: N_2 sorption isotherm for MFM-512 recorded at 77 K. BET surface area = 8.67(13) m²/g.

Table S4: Relationship between water capacity and proton conductivity for selected MOFs in literature.[S5]

MOF*	Water capacity (wt%)	Conductivity (S cm^{-1})	Impedance conditions (RH, Temp)	Reference
Ca(BTC)	22	$1.2E^{-4}$	98%, RT	31
[Zn(L_{Cl})Cl]	12	$4.5E^{-5}$	98%, 31ºC	28
Fe-MIL-53-COOH	10.9	$2.0E^{-6}$	95%, RT	19
$NH_4[MnCr_2(ox)_6]$	9.9	$1.1E^{-3}$	96%, 22 ºC	36
$\{(Zn_{0.25})_8(O)\}Zn_6(L)_{12}$	26	$2.3E^{-3}$	95%, RT	37
Al-MIL-53	7.5	$2.3E^{-8}$	95%, RT	38
SPPO/Fe-MIL-101-NH_2	50.7	$1.0E^{-1}$	98%, RT	39

*BTC^{3-} = benzene tri-carboxylate; L_{Cl} = 3-methyl-2-(pyridin-4-ylmethylamino)butanoic acid, H_8DTMP = diamine-N,N,N′,N′-tetrakis(methylenephosphonic acid), H_8ODTMP = octamethylenediamine-N,N,N′,N′-tetrakis(methylenephosphonic acid) ; ox^{2-} = oxalate, H_2adp = adipic acid; H_2L = 1,3-bis(4-carboxyphenyl)imidazolium.

10. Additional QENS analysis

The dynamics of the protons were probed using the neutron spectrometer IRIS at the ISIS Pulsed Neutron and Muon Source, Chilton, UK; a time-of-flight inverted-geometry crystal analyser spectrometer with diffraction capabilities. In the QENS measurements, neutrons scattered from the sample were energy-analysed by means of Bragg reflections from a single crystal array of pyrolytic graphite close to the backscattering geometry 2θB = 175°, where θB is the Bragg angle of the analyser crystal, and were counted in a detector array covering 27°< 2θ < 158° yielding a wave vector range of 0.4 $Å^{-1}$ to 1.8 $Å^{-1}$. In this study, IRIS was operated in the PG(002) configuration which provides an energy transfer window of -0.5 meV<ħω< 0.5 meV and an energy resolution ΔEres of 17.5 μeV. The powdered sample was loaded into an annular aluminium container having a suitable sample thickness to minimise multiple scattering effects. The QENS data were collected at temperatures between 248 K and 423 K with counting times of 6 h at each temperature. A Vanadium sample measured at 30 K was used for resolution.

The QENS data were treated presuming that a significant fraction of protons in MFM-512 move too slowly for the resolution of the spectrometer meaning they can be assumed to be immobile (*p*) (*i.e.* aromatic protons). The mobile fraction (1-*p*) that is constrained to perform the molecular diffusion consists of the protons coming from the water molecules bound to the metal and the free hydroxy from the carboxylate. The function j_1 the first order spherical Bessel function. Geometrical information of the molecular motions of active protons in MFM-512 were analysed *via* the elastic incoherent structure factor, EISF.

$$EISF = I_{elastic} / (I_{elastic} + I_{QENS})$$

where $I_{elastic}$ and I_{QENS} are the peak intensities of elastic and quasi-elastic scatterings, respectively. To obtain the elastic ($I_{elastic}$) and QENS (I_{QENS}) intensities, the QENS data were fitted to resolution function convoluted with a *Delta* and a *Lorentzian* function as well as a flat background. The EISF data was fitted using the free diffusion inside a sphere model which showed good agreement with the experimental data allowing descriptions of localised diffusive motions to be made.

Table S5: EISF fitting results for MFM-512 at different temperatures

Temperature (K)	P	Standard error
250	0.83141	0.00644
273	0.81032	0.00667
298	0.77671	0.00705
333	0.7347	0.00759
373	0.69691	0.00812
423	0.66555	0.00858
r (Å)	1.8995	0.04871
Reduced Chi-sqr	$1.62395E^{-4}$	
COD (R^2)	0.97295	
Total iterations	6	

11. References

S1. H. Nowell, S. A. Barnett, K. E. Christensen, S. J. Teat, D. R. Allan, *J Synchrotron Radiat.*, 2012, **19**, 435.

S2. (a) G. M. Sheldrick, *SADABS*, empirical absorption correction program based upon the method of Blessing. (b) L. Krause, R. Herbst-Irmer, G. M. Sheldrick, D. Stalke, *J. Appl. Cryst.* 2015, **48**. (c) R. H. Lessing, *Acta Crystallogr.* 1995, **51**, 33.

S3. G. Winter, D. G. Waterman, J. M. Parkhurst, A. S. Brewster, R. J. Gildea, M. Gerstel, L. Fuentes-Montero, M. Vollmar, T. Michels-Clark, I. D. Young, N. K. Sauter, G. Evans, *Acta Cryst.*, 2018, **74**, 85.

S4. (a) G. M. Sheldrick. *Acta Crystallogr.*, 2015, **71**, 3-8. (b) O. V. Dolomanov, L. J. Bourhis, R. J. Gildea, J. A. K. Howard, H. Puschmann, *J. Appl. Cryst*. 2009, **42**, 339.

S5. J. Canivet, A. Fateeva, Y. Guo, B. Coasne and D. Farrusseng, *Chem. Soc. Rev.*, 2014, **43**, 5594.
